# In-depth exploration of software defects and self-admitted technical debt through cutting-edge deep learning techniques

**DOI:** 10.1371/journal.pone.0324847

**Published:** 2025-06-11

**Authors:** Sajid Ullah, M. Irfan Uddin, Muhammad Adnan, Ala Abdulsalam Alarood, Abdulkream Alsulami, Safa Habibullah

**Affiliations:** 1 Institute of Computing, Kohat University of Science Technology, Kohat, Khyber Pakhtunkhwa, Pakistan; 2 College of Computer Science and Engineering, University of Jeddah, Jeddah, Saudi Arabia; 3 Department of Information Technology at Al-kamil, University of Jeddah, Jeddah, Saudi Arabia; 4 Department of Information Systems and Technology, College of Computer Science and Engineering, University of Jeddah, Jeddah, Saudi Arabia; Philadelphia University, JORDAN

## Abstract

Most previous research focuses on finding Self-Admitted Technical Debt (SATD) or detecting bugs alone, rather to addressing the concurrent identification of both issues. These study investigations solely identify and classify the SATD or faults, without identifying or categorising bugs based on SATD. Furthermore, the majority of current methodologies do not incorporate contemporary deep learning techniques. This work presents an innovative method utilising deep learning techniques to discover and classify Self-Admitted Technical Debt (SATD) and to find defects in software comments associated with SATD. The proposed approach detects this issue and classifies and enhances the understanding and localization of defects. The methodology involves developing a deep learning model using diverse data from repositories, including Apache, Mozilla Firefox, and Eclipse. The chosen data set comprises projects, designated SATD examples, and bug instances, facilitating thorough model training and evaluation. The methodology comprises data analysis, preprocessing, and model training utilising deep learning architectures such as LSTM, BI-LSTM, GRU, and BI-GRU, with Transformer models like BERT and GPT-3, in conjunction with machine learning methods. The performance evaluation criteria, such as precision, recall, accuracy, and F1 score, illustrate the efficacy of the suggested method. Comparative assessment with existing methodologies underscores notable improvements, while cross-validation ensures model resilience. All deep learning models achieved an accuracy and precision of 0.98, and transformer models achieved slightly higher metrics. The GPT-3 achieved an overall accuracy of 0.984. We see that using the transfer learning approach the transformer model (GPT-3) outperformed the other as it achieved an overall accuracy of 0.96 and F1-Score of 0.96, precision of 0.96, and recall of 0.96, and deep learning models (LSTM, GRU) also give significant performance, but their accuracy is slightly lower than baseline model (Naive Bayes). The research has significant implications for software engineering, providing a comprehensive method for software quality assessment and maintenance. It enhances software architecture technical debt (SATD) and knowledge of bugs, as well as prioritization and resource allocation for software maintenance and evolution. The research’s ramifications go beyond academia; it has a direct impact on business procedures and makes it easier to create software systems that are reliable and long-lasting.

## 1 Introduction

Software development teams work hard to finish projects on time, under budget, and with high quality. But in practice, quality is occasionally sacrificed to complete the job on schedule. Developers frequently offer short-term remedies or quick fixes, which might not be the best long-term option and could result in the building of technical debt. [1, 2]. Technical debt (TD) is a compromise between the long-term viability of a software system and the immediate advantages of “cutting corners” in software development. As software systems get larger, technical debt tends to rise. When technical debt necessitates more maintenance and development work, it should be managed (at least in part). Self-reported technical debt was divided into many categories by researchers, including design and code debt. Like a loan, interest may accrue if the self-acknowledged technological debt is not paid back right away. Self-admitted technical debt can result from bad coding methods. Prior research has demonstrated the prevalence of self-reported technical debt and its impact on the quality, complexity, and maintainability of software. System changes are more common and more challenging to finish when technical debt is high [3]. The creators understand that TD cannot be prevented and needs to be carefully handled [5]. Software restructuring and redesign serve as two approaches to address technical debt [6]. While managing technical debt is crucial, particularly in the initial stages, its identification can prove challenging. To manage TD properly, it must be comprehensively comprehended after detection. For instance, other engineers on the team may be unaware that a solution is a statement containing a TD if it is executed in code and positioned subsequent to a conditional statement (such as an if statement). The developer includes a comment elucidating the TD for visibility. Such comments indicate an acknowledgment of inherent technological debt [6]. More recent works [7–9] have focused on creating tools to determine if a code comment signals self-acknowledged technical debt. Assume that code fragments with technical debt are accompanied by such comments. However, this assumption is not always true. The use of technical debt in the code is often not explicitly mentioned in the comments. In such instances, it is crucial to implement an automated system that can (i) identify whether a specific code segment constitutes technical debt and (ii) if applicable, generate the suitable comment for integration into the code, contingent upon obtaining consent from the relevant party.Therefore, the proposed system aims to use deep learning techniques to provide a comprehensive method for SATD identification and debt-based technical fault detection. The system takes into account code information and comments, recognizing the importance of comments as an important source of knowledge and understanding. Through the process of training deep neural networks on annotated data sets, the system is able to discover intricate patterns, correlations, and contextual information that are concealed within software code and the observations that accompany it. Enhancing the accuracy and reliability of technical debt and defect identification can be accomplished through the utilisation of advanced natural language processing (NLP) tools, which are utilised to analyse and evaluate feedback in order to derive significant insights. It is possible for the technology to categorise defects and SATDs into a number of different groupings, which provides software developers and businesses with additional alternatives for resolving bugs. The proposed system assists software professionals in making informed decisions and taking proactive measures to mitigate SATDs and defects, offering a comprehensive and intelligent solution that enhances software quality, reduces maintenance costs, and increases the efficiency of the software development process. The primary objective of this project is to design and implement a deep learning system for recognising SATD debt and identifying issues, taking into account both code and comment information. The proposed model acquires knowledge of the correlations among various code patterns, comments, and the occurrence of SATD or defects by training deep learning architectures, including LSTM, BI-LSTM, GRU, and BI-GRU, as well as employing transformer models like BERT and GPT-3, in conjunction with machine learning algorithms on annotated datasets. The objectives of this research study are:

- To propose a cohesive strategy that integrates SATD identification and error detection in software systems, considering the concurrent examination of code and comments.
- To Design and implement deep learning models that leverage advanced neural network architectures to learn patterns, relationships, and contextual information from annotated datasets.
- To investigate and implement natural language processing (NLP) methods for the processing and analysis of comments pertaining to code.
- To conduct extensive experimentation and evaluation of the proposed approach using various software projects and datasets.

In this paper, we investigate the following research questions:

- **RQ1:** How can an integrated approach that combines SATD identification and Bug identification be developed for software systems, considering the simultaneous analysis of code and comments?
- **RQ2:** What is the accuracy and effectiveness of deep learning models utilising advanced neural network architectures in learning relationships, contextual information, and patterns from annotated datasets for the identification of SATD and bugs?
- **RQ3:** How are natural language processing techniques effectively applied to analyze and process the comments associated with code to enhance SATD identification and classification, and Bug identification and classification?
- **RQ4**: What outcomes arise from the comprehensive experimentation and evaluation of the proposed approach utilising various datasets?

RQ1 is designed to explore the feasibility and method of creating a unified approach to simultaneously identify SATD and bugs by analyzing the comments associated with code and classifying them. This approach helps the developers to maintain code quality and effectively manage SATD.RQ2 is designed to investigate the performance of deep learning for identifying SATD, detecting Bugs, and classifying them as compared to baseline models, i.e., traditional machine learning models. RQ3 is designed to explore the role of NLP techniques in analyzing and processing the comments associated with code. By enhancing the SATD identification and Detection through improved comment analysis, this research led to more accurate tools for software maintenance. RQ4 aims to examine comprehensive testing and a proposed integrated approach, evaluating the performance and effectiveness of this approach across various datasets.

### 1.1 Main contributions

This study offers the following key contributions:

- This study presents a new integrated framework that concurrently identifies and classifies Self-Admitted Technical Debt (SATD) and software bugs by utilising code comments and code fragments, areas that have not been previously investigated.
- The proposed system employs advanced deep learning models, including LSTM, Bi-LSTM, GRU, and Bi-GRU, as well as transformer models such as BERT and GPT-3, to effectively capture semantic and contextual relationships between comments and code for accurate classification.
- We apply transfer learning from SATD detection to bug classification, enhancing performance by leveraging shared contextual features across tasks.
- Our research method is validated across diverse real-world datasets (Apache, Eclipse, Mozilla Firefox) with rigorous performance metrics, demonstrating superior results compared to traditional approaches.

## 2 Related work

The proposed system categorises related work into three areas: Self-Admitted Technical Debt, bug identification, and comment generation, as it identifies SATD and bugs through software code and comments.

### 2.1 Self-admitted technical debt

Cunningham was the pioneer in introducing the concept of technical debt, highlighting the importance of balancing short-term objectives, such as software development quality and delivery speed, within the software engineering process [10]. Previous research has demonstrated that technical debt is an unavoidable aspect of software development. If not addressed promptly, it can lead to a decline in product quality and elevate the risk associated with the system [11, 12]. The concept of self-admitted technical debt has recently been introduced, denoting situations where developers explicitly recognise technical debt in code comments or documentation. [14]. This type of technical debt is deliberately created by developers to address the particular requirements of a software project, frequently comprising inferior technological solutions aimed at achieving short-term objectives. These solutions are often prospects for subsequent reworking. Despite the relatively low fraction of self-admitted technical debt (SATD) in a software project, its impact on software quality and maintenance should not be overlooked. The intrinsically unstructured nature of code comments renders rule-based detection methods inefficient, complicating the reliable identification of SATD in source code comments by automated technologies. A recent survey [15] identifies six distinct file-level approaches for the detection of self-admitted technical debt (SATD). The detection methods can be classified into two primary categories: (1) model-based approaches that focus on recognising textual patterns in code comments, and (2) machine learning-based approaches that utilise statistical and linguistic features for SATD classification. In earlier research, the detection of self-admitted technical debt (SATD) primarily relied on text mining techniques to analyze and extract relevant patterns from code comments [16]. Software engineering has increasingly adopted deep learning techniques, leading to substantial advancements across various areas of the field [17].

A proposal and feedback production paradigm for SATD, SADTID, was introduced in a study conducted by [18]. Without depending on pre-existing SATD comments, SATDID searches the source code for cases of technical debt that should be recognized on its own. When technical debt is identified in a code segment, SATDID produces a relevant SATD comment that can be appended to the segment and delineates the debt. To guarantee repeatability and facilitate further research in this domain, code, datasets, and results reports are freely accessible to the public. This research study evaluated the efficacy of SADTID utilising a dataset including code-comment pairings from 4,995 active software development repositories. A comprehensive methodology for the management of SATD was presented by [19] in a separate research study. This framework addresses the lack of code comments. The framework employs specified comments to identify the presence of technical debt. In the absence of a remark, the framework analyses the source code to identify potential technical debt and generates the appropriate SATD comment. The performance of the framework is evaluated using a dataset that includes code-comment pairs from 4,995 active software development repositories, along with a publicly available dataset. The evaluation of the framework encompasses the analysis of three intelligent functions: identification of SATD source code, identification of SATD comments, and creation of SATD comments.

### 2.2 Bug identification

Program analysis is a domain that possesses the capacity to improve software development methodologies. It entails the examination of machine learning models that integrate formal and probabilistic reasoning.The study undertaken [20] progresses in neural information processing systems, namely through BUGLAB, a self-supervised method for learning program analyses. BUGLAB surpasses current methodologies by proficiently identifying defects in practical programs.

### 2.3 Comments generation

A study on deep code comment generation presented a methodology for code summarization, which entails converting programming code into plain language comments. The suggested methodology, termed DeepCom, employs an attention-driven Seq2Seq model. It specifically concentrates on producing method comments in Java. Abstract Syntax Tree (AST) sequences serve as input for DeepCom. The ASTs are transformed into specifically organized sequences via a distinctive Structure-Based Traversal (SBT) approach. SBT maintains the fidelity of the representation by facilitating the depiction of structural information. The experimental results indicate that DeepCom surpasses existing approaches and exceeds machine translation evaluation measures [21]. The proposed system distinguishes itself from existing research by integrating a comprehensive approach to simultaneously identify and classify both Self-Admitted Technical Debt (SATD) and bugs, leveraging advanced deep learning and transformer models. Previous studies have predominantly concentrated on SATD detection or bug identification in isolation, frequently employing static analysis or conventional machine learning methods. In contrast, our approach offers a comprehensive framework that simultaneously tackles both challenges. Furthermore, in contrast to previous methods that rely solely on pattern-based or isolated machine learning techniques, our system utilises an advanced pipeline that incorporates preprocessing, word embeddings, and complex models including LSTM, BiLSTM, GRU, BiGRU, BERT, and GPT-3. This facilitates a more refined comprehension and categorisation of SATD and bugs.

### 2.4 Research gap and motivation

Despite extensive research in SATD detection and bug identification, several key challenges remain unresolved:

- **Disjointed Focus:** Most existing studies focus either on SATD detection or bug classification, but not on their integrated analysis. This disjointed focus limits insights into how SATD correlates with bugs.
- **Outdated Techniques:** Previous approaches primarily rely on static analysis, rule-based detection, or traditional machine learning. These methods often fail to capture nuanced language and contextual cues in comments.
- **Restricted Application of Transfer Learning:** The potential of transfer learning—where knowledge from SATD detection can enhance bug identification—has not been sufficiently explored in prior works.
- **Lack of Holistic Tools:** There is a scarcity of unified models that analyze both code and comments together to improve software maintenance and quality.

To fill these shortcomings, our research suggests a state-of-the-art deep learning system that can detect SATD and bugs at the same time using transfer learning and natural language processing. This system is both comprehensive and practical.

## 3 Architectural framework

**Fig 1:**
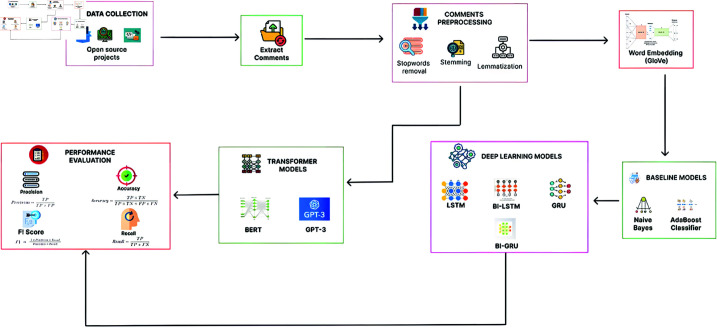
The image illustrates a workflow for processing comments and identifying classifying Self-admitted technical debt.

**Fig 2:**
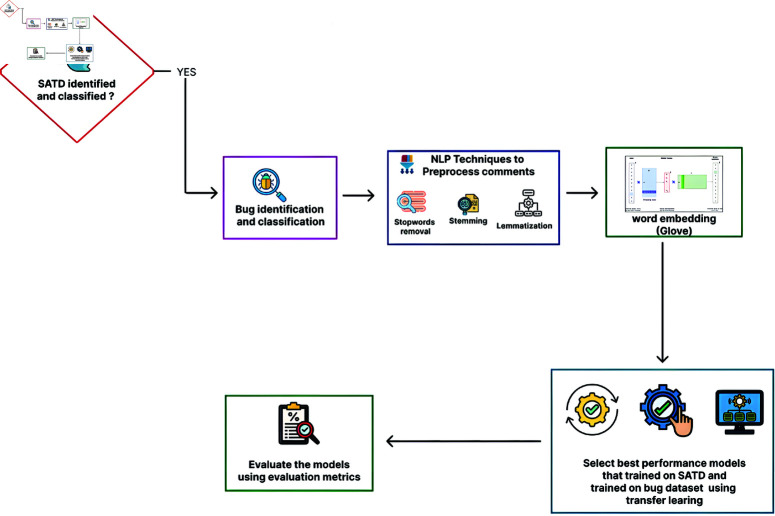
Illustrates Workflow for identifying and classifying bugs based on the basis Self-Admitted technical debt.

The proposed system illustrated in Figs 1 and 2 aims to address the shortcomings of prior research by presenting an integrated approach that concurrently identifies self-admitted technical debt and software bugs.Additionally, it incorporates a comprehensive classification framework to categorize these issues based on their respective types and components.

The system will utilize powerful and advanced deep-learning methodologies to evaluate comments related to software code and assess their cumulative effect on both SATD and bugs. Through the training of machine learning, deep learning, and transformer models on datasets, the system will discern intricate linkages, patterns, and contextual information that aid in the identification of self-reported technical debt and faults based on SATD. The purpose system will offer a cohesive framework for the assessment and classification of technical debt and bugs, allowing software developers and organisations to prioritise and address specific issues based on their components, impact, and potential risk to the system. The integrated approach will offer a holistic view of software quality, facilitating effective decision-making in software maintenance and resource allocation by bridging the gap between SATD and bug identification and providing classification schemes. The proposed system aims to advance the field of software engineering and support the development of robust and sustainable software systems.

The framework’s design comprises numerous elements. The comments extracted from open-source projects undergo preprocessing. Preprocessing of the comments begins with the elimination of stop words (such as “the," “is," and “and") because they lack significant meaning [22]. The selection of this action was made in order to reduce the quantity of data and improve the efficiency of natural language processing tasks such as information retrieval and text categorisation. Then stemming and lemmatization are applied. For example, in stemming the words ‘running,’ ‘runner’ reduces to ‘run’ [23]. For example, in lemmatization, the ‘running’ will be lemmatized to ‘run’; this process ensures the variations of the same word are treated as identical and enhances the analysis accuracy [24].

Word Embedding can also be exploited for the detection of SATD and bugs in comments. Auto-myth prioritization (AMP) through comment analysis creates vectors of self-admitted technical debt and bug-related terms/patterns, using patterns specific to the regex syntax. These embeddings are then used for predicting the existence and amount of self-admitted technical debt in comments as well as detecting areas prone to accumulating further portions of features on which sound diagnostics should be enforced. Words like “code duplication" and “legacy code", which one could argue is among, you know, if a word embedding model trained on the software engineering space to do this would be listed as particularly important terms for technical debt and bugs [25]. For developers, analyzing comments and detecting these kinds of patterns can be helpful to have a better educated guess about the amount of technical debt and bugs their codebase is carrying. Global Vectors for Word Representation (GloVe) is a method for generating word vectors based on co-occurrence statistics [26]. GloVe, or Global Vectors for Word Representation, utilises co-occurrence statistics within a corpus to derive vector representations [27]. The GloVe algorithm starts with a co-occurrence matrix that counts the number of instances each phrase takes place in the same context as every different word in the corpus and then makes use of singular value decomposition (SVD) to generate a co-occurrence matrix no issue, selva2021review). An embedding matrix is constructed. The resulting word vectors are optimized to capture both linear and nonlinear relationships among words based on the order of concurrent words. Subsequently, the identification of SATD is conducted through the application of machine learning models, including Naïve Bayes and AdaBoost Classifier, as well as deep learning models such as LSTM, BI-LSTM, GRU, and BI-GRU, in addition to Transformer models like BERT and GPT-3. When SATD is detected and classified, the next phase is to identify bugs based on SATD by using transfer learning concepts and selecting the best-performing models trained on SATD.

**Fig 3:**
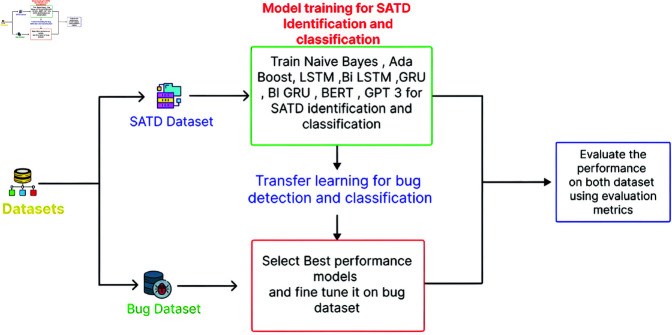
The image illustrates a workflow of identifying and classifying bugs based on self admitted technical debt using transfer learning.

Fig 3 demonstrates the identification and classification of bugs based on SATD utilising a transfer learning methodology. The optimal performance models are chosen from machine learning, deep learning, and transformer models to detect and categorise errors. The transfer learning methodology is subsequently employed to refine the selected model on the bug dataset, followed by an evaluation to assess the performance of each variant.

## 4 Methodology

### 4.1 Technical debt dataset collection and preparation

**Fig 4:**
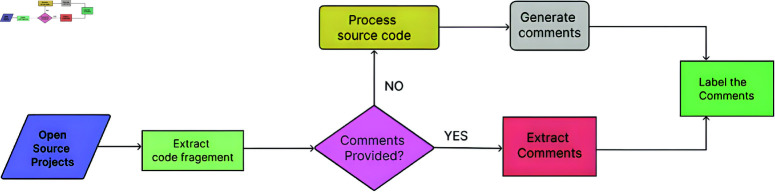
The image Illustrate Workflow for preparing the dataset for identifying and classifying SATD.

The technical debt dataset was carefully selected from a couple of sources to encompass a diverse variety of SATD instances. Sources include software repositories (consisting of Eclipse, Apache, and Mozilla Firefox). Fig 4 shows the complete process of preparing the dataset. First, code snippets were extracted from the above-cited repositories, together with comments and commit messages of seven open-source projects. To ensure quality, the dataset underwent a filtering technique. Irrelevant files (e.g., configuration documents) were excluded, and the simplest code files (e.g., Java, Python, and C++) containing meaningful code snippets and comments were retained. After the filtering process, we check whether comments are provided or not. Most code snippets contain pre-existing comments; therefore, extract these comments. In several code snippets, comments are absent, necessitating a manual analysis of the code to generate corresponding comments for the fragments.

Following the extraction and generation of comments, each comment in the dataset is categorised according to the presence or absence of Self-Admitted Technical Debt (SATD). SATD times are recognized using established criteria, which include keywords in comments consisting of “TODO," “FIXME," or “hack." Also, the comments are labeled on the premise of several types of SATD. The prepared dataset consists of 23,180 samples and three columns of project, text, and type. The project column contains an open-source project from which we extract the code fragments with comments, the text column contains the comments, and the type column consists of different types of SAT, D, also with non-debt type, which shows the absence of SATD in comments.

### 4.2 Bug dataset collection and preparation

The dataset used for Bug identification and classification is the Eclipse bug dataset, which includes reports from the Eclipse platform. The dataset includes numerous attributes associated with bug reports, which include Bug ID, components, Product, and a brief description of each bug. The Eclipse bug dataset was collected from the Bugzilla repository, which is the bug-tracking system used by the Eclipse project. The dataset was downloaded from the Eclipse Bugzilla repository https://bugs.Eclipse.Org/insects/. The data collection process consists of the following steps: Data Retrieval: The bug reports had been retrieved from the Bugzilla repository through the usage of their API and by downloading the information dump supplied by way of the Eclipse project. Data Extraction: The relevant fields (Bug ID, Product, Components, and brief desc) were extracted from the raw data. Data Storage: The extracted data is saved in a standard format, such as a CSV document, for further processing and evaluation. Through the process of collecting and organising the Eclipse bug dataset in this manner, we made certain that the data was prepared for the following preprocessing and machine-learning operations that were to be performed. The created dataset has a total of 60,000 columns, with four columns containing the following information: Bug ID, Components, Product, and Short desc. In the Bug ID column, the unique identifier for each bug is displayed; in the Component column, eleven different components are displayed; in the product column, the project or product that is affected by a defect is displayed; and in the short desc column, a quick explanation of the bug and a concise summary of the issue are presented.

### 4.3 Exploratory Data Analysis (EDA)

Exploratory data analysis is a critical stage in the data analysis process that clarifies underlying patterns, distributions, and correlations.This section provides an exploratory data analysis of the SATD dataset and the bug dataset. Exploratory data analysis provides insights that inform subsequent modelling decisions and strengthen the robustness of our methodology.

#### 4.3.1 SATD dataset.

**Fig 5:**
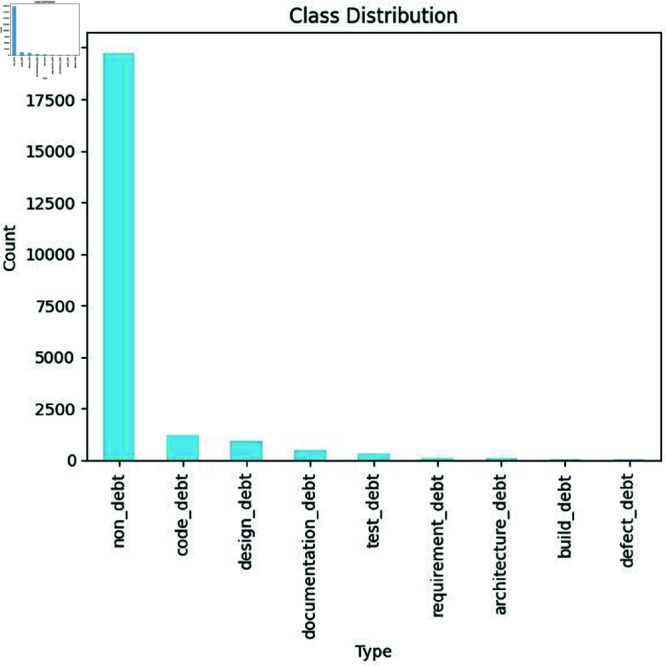
The image Illustrate Distribution of Self-Admitted Technical Debt’s types.

**Fig 6:**
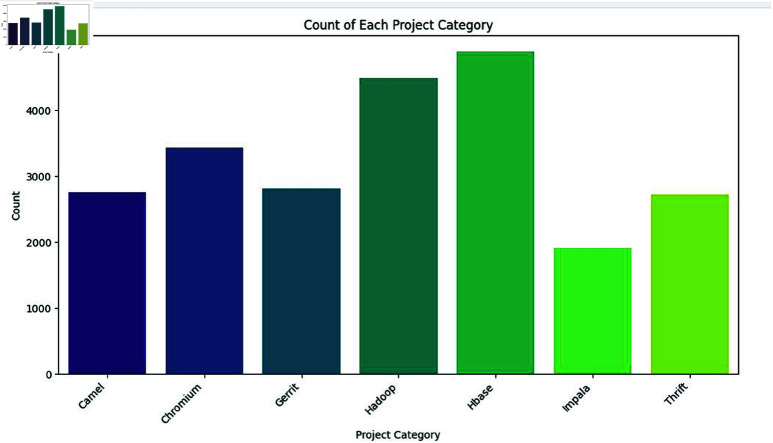
The image Illustrate Count of instances for each project category.

**Fig 7:**
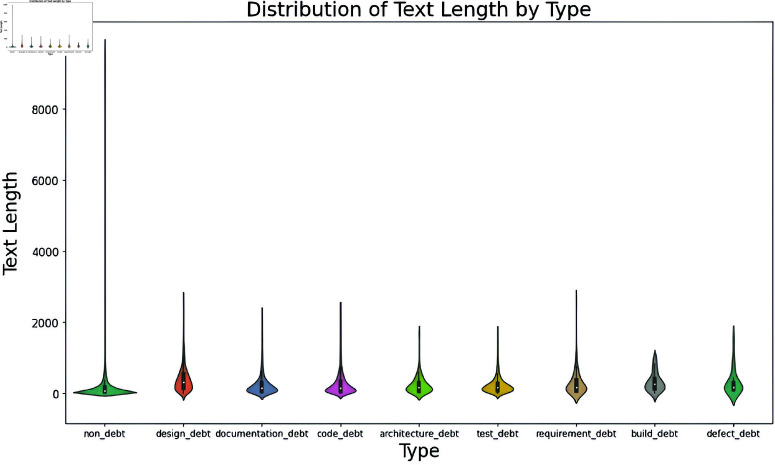
The image Illustrate distribution of Text Length Across Different Types of SATD.

- **Class distribution of SATD types:** The distribution of various SATD types within a dataset is illustrated in Fig 5. As we see in the graph the non-debt category has the highest number of instances about 19759, code debt has about 1246 instances, the design debt bar shows it has 935 numbers of instances present in the dataset, documentation debt has about 486 numbers of instances, test debt has 438 instances but the requirement debt, architecture debt, build debt, defect debt have very few instances in the dataset with the count of 96, 87, 64, and 25. This distribution shows a high-class imbalance problem within the dataset, where a large number of instances belong to the non-debt category.

- **Count for each project category:** Fig 6 shows the count of instances for each category of project within the dataset. We have collected the comments associated with the code from the open-source project. The seven open-source projects where the comments were gathered are clearly displayed in this graph: Camel, Chromium, Gerrit, Hadoop, Hbase, Impala, and Thrift. The graph shows the count of instances for each project, we see that Hbase and Hadoop have the highest count, with over 4500 instances for each, indicating these two projects are the most frequent in the dataset. Chromium also has the highest counts, while Camel, Gerrit, and Thrift have moderate counts, and Impala has the lowest counts. This helps in understanding that most categories are more prevalent than others.

- **Text length distribution by Type column:** Fig 7 shows the text length variations across different types. The plot reveals that non-debt entries have a significantly wider range of text length, with some text lengths exceeding 10000 characters. On the other hand, other types of debt, such design, documentation, code, architecture, test, requirement, build, and defect, tend to cluster around shorter text lengths and have a more enclosed distribution. This analysis helps us to understand that some outliers exist, particularly in the non-debt category. Also, this visualization helps us to understand the textual characteristics associated with each type of debt, indicating that non-debt entries are more verbose. This is due to the imbalanced distribution of different types of SATD.

#### 4.3.2 Bug dataset.

**Fig 8:**
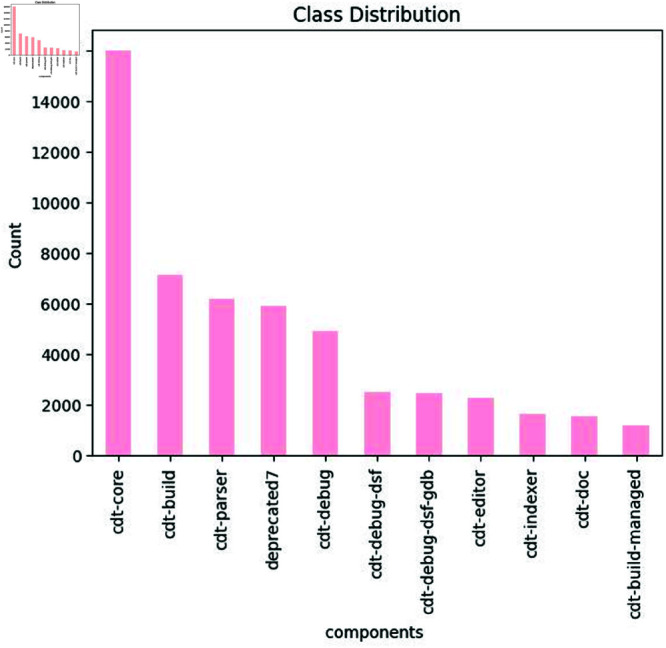
The image Illustrate distribution of different bug components.

**Fig 9:**
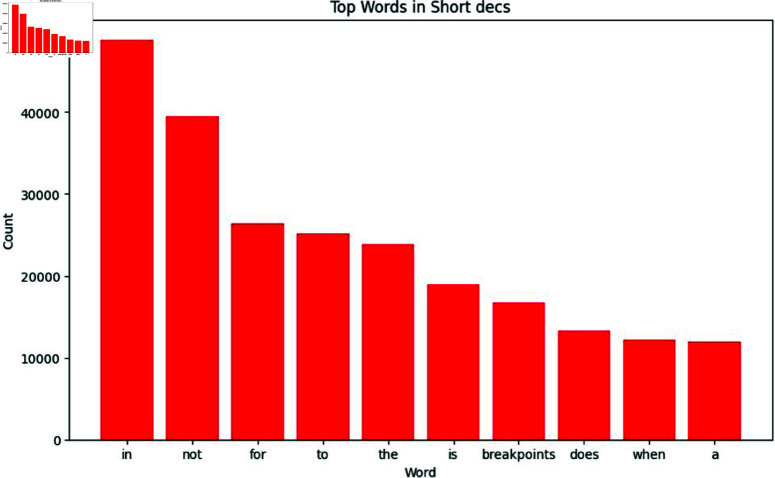
The image Illustrate Visualization of most common terms to understand stop words in short desc.

- **Class distribution of different bug components**: Fig 8 demonstrates how the various components of the dataset are distributed across the entire dataset. The components are shown along the x-axis, and the count of each component is shown along the y-axis in the graph. cdt-parser, deprecated, cdt-build, cdt-core, cdt-debug, cdt-doc, cdt-indexer, cdt-debug-]dsf, cdt-debug-dsf-gdb, cdt-build-managed, and cdt-editor are some of the components that are included. As we see in the graph distribution, each component has a different count. A Cdt-core component has the highest number of instances, about 15995. In contrast, cdt-parser, deprecated7, cdt-build, cdt-debug, cdt-doc, cdt-indexer, cdt-debug-dsf, cdt-debug-dsf-gdb, cdt-build-managed, and cdt-editor have 7147, 6165, 592, 4926, 2505, 2505, 2471, 2286, 1624, 1526, 1198 numbers of instances, respectively. This reveals the imbalance problem in the distribution of different components. We need to address this imbalance problem to classify the bug based on different components.

- **Visualization of Top terms in comments:** Fig 9 shows the bar chart that represents the top terms in comments represented by the column “short desc". The most common word is “in," which appears about 50,000 times, and other words like not, for, to, and each occur between 20,000 and 40,000 times. Key terms include “breakpoint”, “does”, “when,” and “a”. The higher frequency of stop words indicates that these are frequently used in comments or short descriptions. This visualization helps us to understand the stop words used in the dataset, we remove these stop words in text preprocessing.

### 4.4 Data preprocessing

#### 4.4.1 Technical debt dataset.

**Fig 10:**
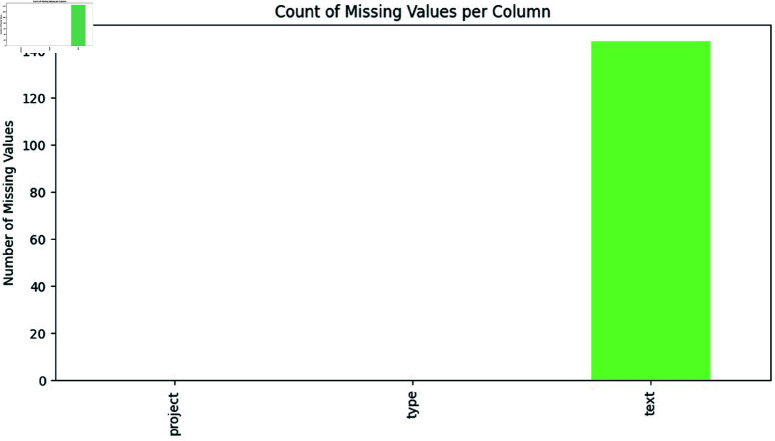
The image Illustrate missing values in SATD dataset.

**Fig 11:**
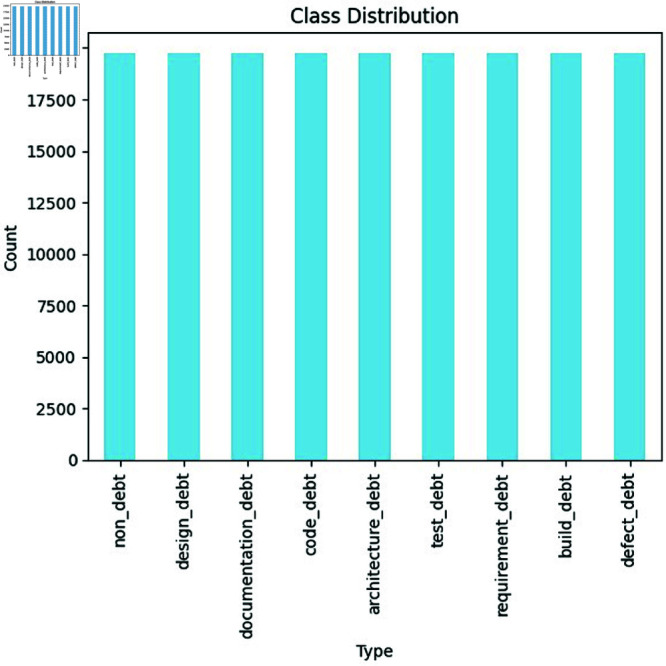
The image Illustrate class distribution of different types of SATD after balancing the dataset using SMOTE-TEXT technique.

The dataset preprocessing includes the following steps:

- **Handle missing values**: We identified 144 missing values in the dataset, as shown in Fig 10. Initially, we considered several strategies for handling these missing values, including imputation methods such as mean, median, or mode imputation. However, due to the nature of the dataset and the proportion of missing values, we decided to use dropna() to remove rows with missing values. This approach ensures that only complete records are retained for further processing, although it may result in some loss of data. The decision to use dropna() was based on the need to maintain data integrity and minimize bias in the dataset.
- **Check for class distribution:** We conducted a thorough analysis of the class distribution within the dataset to assess the extent of class imbalance. The distribution of classes was found to be highly imbalanced, as detailed below in Table 1.
  This imbalance could potentially affect model performance, leading to biased results.
- **Balance the dataset:** To address the class imbalance, we applied the Synthetic Minority Over-Sampling Technique for Text (SMOTE-TEXT). SMOTE-TEXT generates synthetic instances to balance class distributions, thereby improving model performance by providing a more representative dataset. We chose SMOTE-TEXT due to its effectiveness in handling textual data and its ability to create realistic synthetic samples. Parameter Tuning: We configured SMOTE-TEXT with parameters such as k-neighbors and sampling strategy, performing cross-validation to determine the optimal settings. This ensured that the synthetic samples generated were representative of the minority classes while preserving data integrity. Validation and Impact Assessment: After applying SMOTE-TEXT, we evaluated the effectiveness of the balancing process by assessing class distribution post-processing. Fig 11 shows the balanced distribution of different types after applying the SMOTE-TEXT technique.

**Table 1:** Class distribution of different types of technical debt.

Type of SATD	Number of Instances
Non-Debt	19,759
Code Debt	1,246
Design Debt	935
Documentation Debt	486
Test Debt	338
Requirement Debt	96
Architecture Debt	87
Build Debt	64
Defect Debt	25

#### 4.4.2 Bug dataset.

**Fig 12:**
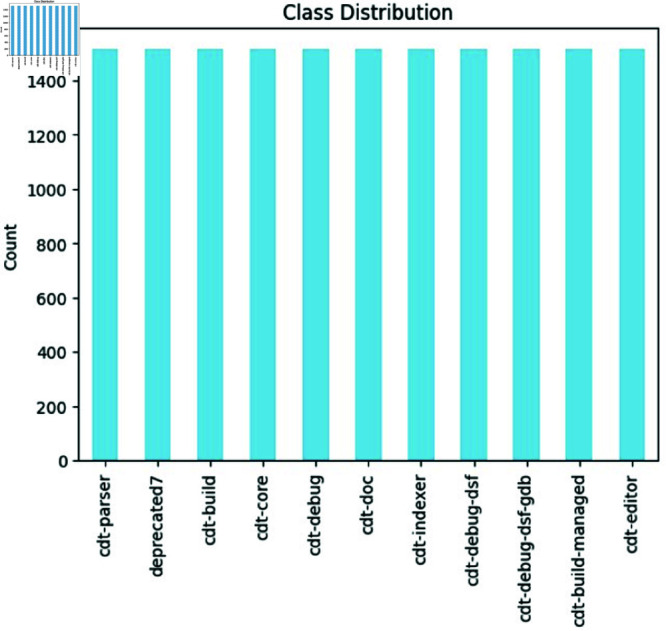
The image Illustrate class distribution of different bug components after balancing the dataset using SMOTE-TEXT technique.

Bug dataset preprocessing includes the following steps:

- **Handling missing values:** A comprehensive inspection was performed to detect any missing values within the bug dataset. The analysis confirmed that the dataset is complete, with no missing entries, ensuring its suitability for subsequent analysis.
- **Check for class distribution:** We assessed the class distribution within the bug dataset to identify any imbalances among the classes. The distribution was found to be highly imbalanced, with some classes being significantly underrepresented compared to others.The detailed below in Table 2.
- **Balance the dataset:** We utilised the Synthetic Minority Over-sampling Technique for Text (SMOTE-TEXT) to rectify the imbalance in class distribution. This technique produces synthetic instances for minority classes, thereby achieving dataset balance. SMOTE-TEXT was applied to create synthetic bug instances for underrepresented classes. This method ensures that the dataset includes a more balanced representation of each class, which is crucial for training robust machine learning models. After applying SMOTE-TEXT, the dataset achieved a more balanced distribution of bug components. This adjustment helps in improving the performance of classification models by ensuring that each class is adequately represented during training. Fig 12 shows the balanced distribution of different bug components after balancing the dataset. This visualization illustrates the effectiveness of the balancing technique in equalizing class representation.

**Table 2:** Class distribution of different bug components in the dataset.

Bug Component	Number of Instances
CDT-Core	15,995
CDT-Build	7,147
CDT-Parser	6,165
Deprecated7	5,921
CDT-Debug	4,926
CDT-Debug-DSF	2,505
CDT-Debug-DSF-GDB	2,471
CDT-Editor	2,286
CDT-Indexer	1,624
CDT-Doc	1,526
CDT-Build-Managed	1,198

### 4.5 Feature extraction

To enhance the quality of both datasets, we applied feature extraction techniques to the textual data. This includes:

#### 4.5.1 Text preprocessing

Preprocessing of the comments begins with the elimination of stop words (such as “the,” “is,” and “and”) because they lack significant meaning [22]. This measure was implemented to reduce data volume and improve the efficacy of NLP tasks such as information retrieval and text classification. Subsequently, stemming and lemmatisation are executed to convert words to their base or root form. For instance, the terms ’running’ and ’runner’ are reduced to ’run’ through stemming [23]. For example, in lemmatization, the ‘running’ will be lemmatized to ‘run’; this process ensures the variations of the same word are treated as identical and enhances the analysis accuracy [24].

#### 4.5.2 Data vectorization

Text data is often extremely sparse and subject to the challenge of high dimensionality. When employing one-hot encoding to represent words as vectors, each word is mapped to a vector whose length equals the full vocabulary size, containing zeros in every position except for a single one indicating the word’s specific index within the vocabulary’s dataset [28]. The word here refers to textual comments in the dataset. To elevate this problem, we used the technique called GloVe (Global vector for word representation). We used this technique for converting textual data into numerical vectors. GloVe is a popular unsupervised learning algorithm for obtaining vector representation for words [29]. It maps the words into a continuous vector space based on the word co-occurrence statistics from a large corpus of text. To implement GloVe, we utilized the pre-trained GloVe embedding available at Stanford NLP Group, trained on a dataset of 6 billion tokens. The GloVe pre-trained embeddings provide a fixed-length vector representation for each vocabulary word, and then apply GloVe on preprocessed text. A set of vectors, each representing a word in the input text, forms the final Embedding Matrix. The deep learning models were subsequently able to process and learn from the textual data by using these vectors as input features. The actual words or tokens are not accessible to the implementation; it only works with these indices and their vector representations.

## 5 Experimental setup

In this section,initially, we provide an overview of our models for both datasets, which includes baseline models, deep learning models, and transformer models. We also provide an explanation of the model selection procedure for the bug dataset. We present the parameter setting for both datasets following the introduction of models and conclude by providing a detailed explanation of the evaluation procedure employed in our experiments.

### 5.1 Model section for self-admitted technical debt

#### 5.1.1 Baseline models

For baseline models, we selected the Naive Bayes and AdaBoost classifiers due to their effectiveness in classification tasks and their simplicity and interpretability.

1. **Naive Bayes Classifier:** Naive Bayes is a supervised machine learning algorithm used for classification tasks such as text classification. The naive Bayes classifier is based on the Bayes theorem. Naive Bayes used the principle of probability to perform the classification task [30]. Bayes’ theorem is expressed as:
  $$P(Y|X)=\frac{P(X|Y)\cdot P(Y)}{P(X)}$$ (1)

where:

- P(Y∣X) is the posterior probability of *Y* given *X*.
- P(X∣Y) is the likelihood of *X* given *Y*.
- *P*(*Y*) is the prior probability of *Y*.
- *P*(*X*) is the marginal probability of *X*.

1. **AdaBoost Classifier:** Brief overview of adaptive boosting is that the AdaBoost classifier is an ensemble machine learning technique employed for diverse tasks classification. The AdaBoost classifier is a supervised technique of machine learning that enhances classification by amalgamating several weak learners into more robust learners [31] The AdaBoost algorithm can be described with the following equations:
  1. The weighted error of the *t*-th weak classifier *h*_*t*_(*x*):
    $$\epsilon_{t}=\frac{\sum_{i=1}^{n}w_{i}^{\left(t\right)}I(y_{i}\neq h_{t}(x_{i}))}{\sum_{i=1}^{n}w_{i}^{\left(t\right)}}$$ (2)
  2. The weight update rule for the weak classifiers:
    $$\alpha_{t}=\frac{1}{2}\ln\left(\frac{1-\epsilon_{t}}{\epsilon_{t}}\right)$$ (3)
  3. The update rule for the sample weights:
    $$w_{i}^{\left(t+1\right)}=w_{i}^{\left(t\right)}\exp\left(-\alpha_{t}y_{i}h_{t}(x_{i})\right)$$ (4)
  4. The final strong classifier *H*(*x*):
    $$H(x)=\text{sign}\left(\sum_{t=1}^{T}\alpha_{t}h_{t}(x)\right)$$ (5)

#### 5.1.2 Deep learning models.

We chose the LSTM, BI-LSTM, GRU, and Bi-GRU as deep learning models. The rationale for choosing these models is in their efficacy in handling textual and sequential data, particularly in natural language processing applications. We implement customisation for all deep learning models to enhance their efficacy. The modifications implemented on deep learning models are elucidated further in the document.

1. **LSTM (Long Short-Term Memory Model):** LSTM represents a specific kind of RNN that addresses the vanishing gradient issue found in conventional RNN architectures. LSTM effectively addresses vanishing gradient issues, making it well-suited for tasks in natural language processing. The system incorporates a memory cell designed to preserve information across an extended sequence. This approach effectively captures the long-term dependencies crucial for comprehending the context of SATD in comments [32].

**Algorithm 1. An algorithm for LSTM.**

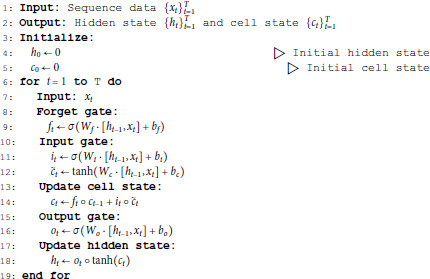



Algorithm 1 illustrates the operation of an LSTM model. At Line 1, the algorithm accepts a sequence of data $x_{t}{t=1}^{T}$, where each *x*_*t*_ represents the input at time step *t*. Line 2 outlines the output, including the sequences of hidden states $h_{t}{t=1}^{T}$ and cell states ${c_{t}}_{t=1}^{T}$. Initialization at Lines 3 to 4 sets the initial hidden state *h*_0_ and cell state *c*_0_ to zero, establishing the network’s initial memory state. Iterating from Line 5 through Line 12, the algorithm processes each time step *t* from 1 to *T*.

At Line 6, input *x*_*t*_ is processed. Line 7 calculates the forget gate *f*_*t*_ using the sigmoid function $f_{t}\;\leftarrow \;\sigma (W_{f}\;\cdot \;[h_{t-1},x_{t}]\;+\;b_{f})$, determining how much of the previous cell state *c*_*t*−1_ to retain. Lines 8 and 9 compute the input gate *i*_*t*_ and the candidate cell state $\tilde{c}t$ as $i_{t}\;\leftarrow \;\sigma (W_{i}\;\cdot \;[ht-1,x_{t}]\;+\;b_{i})$ and $\tilde{c}t\;\leftarrow \;\tanh(W_{c}\;\cdot \;[ht-1,x_{t}]\;+\;b_{c})$, respectively, updating the cell state with new information. Line 10 updates the current cell state *c*_*t*_ using $c_{t}\;\leftarrow \;f_{t}\;\circ \;c_{t-1}\;+\;i_{t}\;\circ \;\tilde{c}t$, where ∘ denotes element-wise multiplication. Line 11 computes the output gate *o*_*t*_ as $o_{t}\;\leftarrow \;\sigma (W_{o}\cdot [ht-1,x_{t}]\;+\;b_{o})$, deciding how much of the cell state contributes to the hidden state. Finally, Line 12 updates the hidden state as $h_{t}\;\leftarrow \;o_{t}\;\circ \;\tanh(c_{t})$, integrating memory and output signals for the LSTM cell at the current time step. This iterative process enables the LSTM to selectively retain or forget past information, crucial for capturing long-term dependencies in sequential data.

2. **Bidirectional LSTM (BiLSTM)**: The bi-directional long short-term memory model is intended to analyse information in both reverse and forward directions. A Bi-LSTM is distinguished from a standard LSTM by its bidirectional input flow. In LSTM, the input advances in a unidirectional fashion, however in Bi-LSTM, the input moves in both forward and backward directions to retain knowledge from both future and past contexts [33] Bi-LSTM analyses sequential data by employing two separate LSTM networks. Every LSTM unit consists of three gates: the input gate, the output gate, and the forget gate. These gates control the flow of information dissemination. The forward LSTM analyses the sequence in a linear progression from beginning to end. In contrast, the backward LSTM analyses the information or sequence from the end to the beginning, and subsequently, the outputs from both networks are combined to produce the final prediction. The Bi-LSTM is often utilised in natural language processing tasks, such as text categorisation, due to its ability to effectively capture the contextual relationships between words and sentences [34]. The Bi-LSTM adopted the LSTM algorithm as:

**Algorithm 2. An algorithm for Bi-LSTM.**

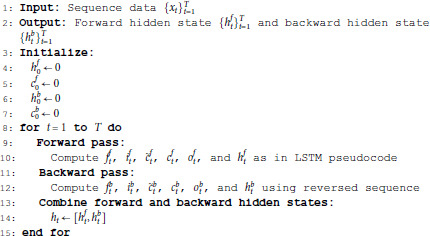



Algorithm 2 describes the functioning of a Bidirectional LSTM (BiLSTM) model. Initially, at Line 1, the algorithm receives an input sequence $x_{t}{t=1}^{T}$, where *x*_*t*_ is the input at each time step *t*. The outputs, specified in Line 2, are two sequences of hidden states: the forward hidden states $h_{t}^{f}{t=1}^{T}$ and the backward hidden states ${h_{t}^{b}}_{t=1}^{T}$, reflecting the dual directional processing of the model.

Lines 4 to 7 handle the initialization of the hidden and cell states for both directions. The forward LSTM is initialized with $h_{0}^{f}$ and $c_{0}^{f}$ set to zero, while the backward LSTM starts with $h_{0}^{b}$ and $c_{0}^{b}$ also set to zero. Beginning at Line 8, the algorithm iterates through the sequence, with Line 9 initiating the forward pass. During this pass, Line 10 computes the forget gate $f_{t}^{f}$, input gate $i_{t}^{f}$, candidate cell state ${\tilde{c}}_{t}^{f}$, updated cell state $c_{t}^{f}$, output gate $o_{t}^{f}$, and hidden state $h_{t}^{f}$, all using the standard LSTM formulas.

Line 11 begins the backward pass, and in Line 12, the backward LSTM calculates the corresponding gates and states ($f_{t}^{b}$, $i_{t}^{b}$, ${\tilde{c}}_{t}^{b}$, $c_{t}^{b}$, $o_{t}^{b}$, $h_{t}^{b}$) but processes the sequence in reverse order (from *T* to 1). In Line 13, the two directional hidden states are integrated, and Line 14 combines them through concatenation as $h_{t}\;\leftarrow \;[h_{t}^{f},h_{t}^{b}]$, creating a unified representation for each time step.

This bidirectional architecture enables the model to capture both past and future context within the sequence, making it highly suitable for tasks such as natural language processing, where understanding the full sequence is crucial.

3. **Gated Recurrent Unit (GRU):** Gated Recurrent Unit (GRU) architecture is a variant of recurrent neural networks (RNN), similar to LSTM (Long Short-Term Memory), and is specifically designed to capture long-term dependencies in sequential data. GRU possesses a reduced number of parameters compared to LSTM and requires less computational resources. The system is composed of gates, specifically the reset gate and the update gate. The reset gate controls how much of the previous hidden state is discarded, whereas the update gate regulates the extent of new input used to modify the hidden state [35]. GRU may be used in various sequential data modeling tasks, including natural language processing; its less complicated structure makes it faster to train. The GRU (Gated Recurrent Unit) cell adopted LSTM algorithm as:

**Algorithm 3. An algorithm for GRU.**

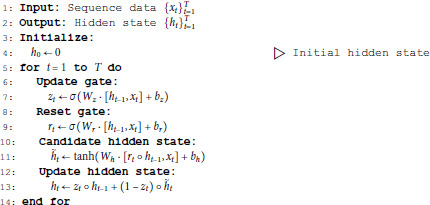



The Algorithm 3 shows the working of the GRU model. In Line 1, the algorithm accepts a sequence of input data $\{x_{t}{\}}_{t=1}^{T}$, where *x*_*t*_ represents the input vector at time step *t*. Line 2 defines the output as a sequence of hidden states $\{h_{t}{\}}_{t=1}^{T}$ generated by the GRU model across all time steps. In Line 4, the hidden state *h*_0_ is initialized to zero. This acts as the starting memory for the network before processing any inputs. From Line 5 onward, the algorithm enters a loop iterating over each time step *t* from 1 to *T*. Line 6 marks the beginning of the update gate computation. In Line 7, the update gate *z*_*t*_ is calculated as $z_{t}\;\leftarrow \;\sigma (W_{z}\;\cdot \;[h_{t-1},x_{t}]\;+\;b_{z})$, where σ denotes the sigmoid activation function. This gate determines how much of the previous hidden state should be retained. Lines 8 and 9 handle the reset gate. The reset gate *r*_*t*_ is computed as $r_{t}\;\leftarrow \;\sigma (W_{r}\;\cdot \;[h_{t-1},x_{t}]\;+\;b_{r})$, which controls how much of the past information to forget when computing the candidate hidden state. In Lines 10 and 11, the candidate hidden state ${\tilde{h}}_{t}$ is calculated using the reset-modulated hidden state from the previous step: ${\tilde{h}}_{t}\;\leftarrow \;\tanh(W_{h}\;\cdot \;[r_{t}\;\circ \;h_{t-1},x_{t}]\;+\;b_{h})$, where ∘ denotes element-wise multiplication and tanh is the hyperbolic tangent activation function. Finally, Lines 12 and 13 update the hidden state *h*_*t*_ as a linear interpolation between the previous hidden state *h*_*t*−1_ and the candidate hidden state ${\tilde{h}}_{t}$, weighted by the update gate: $h_{t}\;\leftarrow \;z_{t}\;\circ \;h_{t-1}\;+\;(1\;-\;z_{t})\;\circ \;{\tilde{h}}_{t}$. This blending mechanism allows the GRU to control the flow of information and adaptively capture both short-term and long-term dependencies in the input sequence.

4. **Bidirectional GRU (BiGRU):** A Bi-directional GRU, abbreviated as Bi-GRU, is a sequence processing model comprising two GRUs. One GRU processes information in the forward direction, while others process data in the reverse way citation zhang2023text. It is a bidirectional recurrent neural network with solely input and forget gates. Furthermore, it integrates the computational efficiency of GRU, rendering it suitable for applications that include sequential data [36]. The BiGRU adopted the LSTM algorithm.

**Algorithm 4. An algorithm for Bi-GRU.**

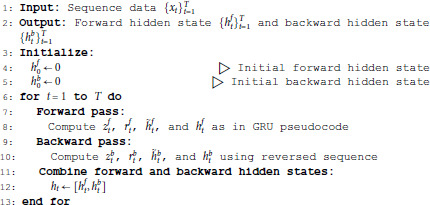



The Algorithm 4 shows the working of the BiGRU model. In Line 1, the algorithm receives a sequence of input vectors $\{x_{t}{\}}_{t=1}^{T}$, where each *x*_*t*_ corresponds to the input at time step *t*. Line 2 defines the outputs of the model: a set of forward hidden states $\{h_{t}^{f}{\}}_{t=1}^{T}$ and a set of backward hidden states $\{h_{t}^{b}{\}}_{t=1}^{T}$, representing the outputs from processing the sequence in both directions. Lines 4 and 5 initialize the forward hidden state $h_{0}^{f}$ and backward hidden state $h_{0}^{b}$ to zero vectors, providing a neutral starting point for memory in both directions of the network. From Line 6 onward, the algorithm iterates over each time step *t* from 1 to *T*. Line 7 begins the forward pass, and Line 8 performs the standard GRU computations: the update gate $z_{t}^{f}$, reset gate $r_{t}^{f}$, candidate hidden state ${\tilde{h}}_{t}^{f}$, and final hidden state $h_{t}^{f}$ are all calculated using the formulas defined in the GRU model, based on the current input *x*_*t*_ and the previous forward hidden state $h_{t-1}^{f}$. Line 9 starts the backward pass, and in Line 10, a similar GRU computation is performed but on the reversed input sequence. The backward update gate $z_{t}^{b}$, reset gate $r_{t}^{b}$, candidate hidden state ${\tilde{h}}_{t}^{b}$, and hidden state $h_{t}^{b}$ are computed by processing the sequence from the opposite direction, allowing the model to capture future context information. In Line 11, the algorithm combines both directional hidden states to form a unified representation at each time step. Line 12 executes this by concatenating the forward and backward hidden states: $h_{t}\leftarrow [h_{t}^{f},h_{t}^{b}]$. This combined representation benefits from both past and future contextual information, making the Bi-GRU particularly effective for tasks such as sequence labeling, language modeling, and other applications in natural language processing.

#### 5.1.3 Transformer models.

1. **Bidirectional Encoder Representations from Transformers (BERT):** Google’s BERT model, which is built on transformers, has surpassed all previous efforts in numerous NLP tasks. In order to train a deep directional representation of text, BERT employs the transformer architecture. All of the layers of this representation are trained concurrently using left and right contexts. The BERT embedding is able to pick up on the text’s contextual relationships and rich semantic information. Classification of texts and language understanding are two areas where it excels [37].
2. **Generative Pre-trained Transformer 3 (GPT-3):** GPT-3 represents a significant advancement in autoregressive language modeling, developed by OpenAI, featuring an impressive 175 billion parameters. The model, built on transformer architecture, underwent training through unsupervised learning utilizing an extensive collection of text data. This system produces text that is contextually appropriate in response to a specified prompt, making it ideal for tasks involving natural language processing and generation [38].

### 5.2 Models selection process for bug identification and classification

We employed the transfer learning strategy described in Sect 3 (Architectural Framework) above to identify and categorise the bugs according to SATD. In order to choose models for the bug dataset, we first assess how well models trained on the SATD dataset perform, choose the top-performing model, and then train that model on the bug dataset. Later in the study, the performance measurements are described in more detail. Following the models’ review, we choose the models shown below.

- **Naïve Bayes:** As the baseline model
- **LSTM and GRU:** As the deep learning models
- **GPT-3:** As the transformer model We trained and evaluated the performance of these pre-trained models on the bug dataset.

### 5.3 Parameter settings

#### 5.3.1 For self-admitted technical debt.

All the parameters we used for training all the models are shown in Table 3. Now we explain all the parameters in detail: The Baseline models were trained using the following parameter settings:

**Table 3:** Training Parameters Setting for Baseline, Deep Learning, and Transformer Models on SATD Dataset.

Model	Epochs	Batch Size	Learning Rate	Dropout Rate	Early Stopping Patience	Fine-Tuning
**Gaussian Naive Bayes**	N/A	N/A	N/A	N/A	N/A	Prior Probabilities: determined from training data; Variance Smoothing: 1e-9
**AdaBoost**	N/A	N/A	0.1	N/A	N/A	Number of Estimators: 1000; Base Estimator: decision stumps
**LSTM**	7	128	0.001	0.5	2	9 dense layers with ReLU, softmax for the final layer, and attention mechanism
**GRU**	7	128	0.001	0.5	2	9 dense layers with ReLU, softmax for the final layer, and attention mechanism
**BiLSTM**	7	256	0.001	0.5	2	9 dense layers with ReLU, softmax for the final layer, and attention mechanism
**BiGRU**	7	256	0.001	0.5	2	9 dense layers with ReLU, softmax for the final layer, and attention mechanism
**BERT**	7	16	2e-5	0.1	2	Add dense layers with ReLU activation on top of the pre-trained BERT model for fine-tuning to enhance non-linearity and learning capacity..
**GPT-3**	7	32	1e-5	0.1	2	Use precise prompts to classify text into 9 classes with GPT-3, setting temperature below 0.2 for consistency.

Naive Bayes classifier trained with its default parameters, including Prior probability, which specifies the prior probabilities of the classes. By default, it is set to zero to ensure that the prior is determined from training data, and the other parameter is variance smoothing, which controls the smoothing applied to variance estimate we used it with a default value of 1e-9 to avoid numerical instability. AdaBoost classifiers are trained using the following parameter settings: Number of estimators (n-estimators) sets to 1000, which means that AdaBoost models were composed of 1000 weak classifiers and learning rate set to 0.1 and by default as based estimators AdaBoost used decision stumps as a weak classifier. All deep learning models while trained using the following parameter settings: Adam optimizer with default parameter was used for training of all models to mitigate overfitting. Dropout layers with a rate of 0.5 were integrated into the models during the training process to avoid overfitting and save computational resources. We used early stopping with a patience of 2 to halt the training when validation loss ceased improving. The numbers of epochs refer to the number of times the entire data set is passed forward and backward through the neural network. So, for all the deep learning models, the number of epochs is 7 by using early stopping to mitigate the overfitting problem. For all the deep learning models, we added 9 dance layers, which align with nine unique classes in the dataset. The dense layers with RELU functions were added to capture the complex pattern in the dataset. The final dense layer utilized the softmax activation function to identify and classify the comments into one of 9 unique classes. Furthermore, an attention mechanism was integrated to enable the model to concentrate on the most pertinent segments of the input sequence, so enhancing the comprehension of contextual information. This architecture of all models facilitates the learning of hierarchical representations of input data and boosts the model’s capacity to accurately recognise and categorise instances into appropriate categories. The batch size denotes the quantity of samples processed before to the update of the model’s internal parameters. A batch size of 128 is utilised for LSTM and GRU, while 256 is utilised for BI-LSTM and BI-GRU, optimising computational efficiency and improving model performance. The transformer models were trained with the subsequent parameter configurations: BERT was trained using a batch size of 16, employing early stopping with a patience of 2, a learning rate of 2e-5, and a dropout layer with a rate of 0.1. The epoch count for BERT is 7. We incorporate supplementary dense layers atop the pre-trained BERT model for the purpose of fine-tuning. We employed the ReLU (Rectified Linear Unit) activation function in these thick layers to incorporate non-linearity and enhance learning capacity. GPT-3 was trained for 7 epochs with a batch size of 32, a learning rate of 1e-5, and a dropout layer with a rate of 0.1. Early halting is employed with a patience parameter of 2. We employed Craft prompts that explicitly direct GPT-3 to generate or categorise text into one of the nine classifications. Furthermore, adjust the temperature to below 0.2 for enhanced classification accuracy.

### 5.4 For bug dataset

First, we explained the process of transfer learning, and then we presented the parameter settings for the bug dataset. All the parameters are shown in Table 4.

**Table 4:** Training parameters setting for pre-trained Baseline, Deep Learning, and transformer Models on Bug Dataset.

Model	Epochs	Batch Size	Learning Rate	Dropout Rate	Early Stopping Patience	Fine Tuning
**Pre-trained Naive Bayes**	N/A	N/A	N/A	N/A	N/A	Prior Probabilities: determined from training data; Variance Smoothing: 1e-9
**Pre-trained LSTM**	8	128	0.001	0.5	2	11 dense layers with ReLU, softmax for the final layer, and attention mechanism
**Pre-trained GRU**	8	128	0.001	0.5	2	11 dense layers with ReLU, softmax for the final layer, and attention mechanism
**Pre-trained GPT-3**	12	32	1e-5	0.1	2	Uses Craft prompts that clearly instruct GPT-3 to generate or classify text into one of the 11 classes. Also, set the temperature to lower than 0.1 for better classification. Add a new output layer.

The baseline model for Bug identification and classification is Naive Bayes, which is trained on SATD. We used this pre-trained baseline model for Bug identification and classification using a transfer learning approach. The process of applying transfer learning involves leveraging a pre-trained model to improve performance on a new but related task. Since the Naive Bayes model is inherently probabilistic it does not require a meaningful change in architecture but instead, we focus on transferring the knowledge through the feature representation. For this bug dataset was reprocessed and transformed into GloVe embedding like the SATD dataset. The text data was tokenized and converted into a vector using the GloVe embedding matrix. We ensured that the features used for the bug dataset were consistent with those used for the SATD dataset, this consistency enabled the model to leverage the learned representation effectively. When features are transformed using a pre-trained model with the help of transfer learning, we fine-tune the model on the bug dataset fine-tuning involves adjusting the model parameter based on the new data set that allows the model to learn specific patterns relevant to the Bug identification. We used the default parameter as used in SATD data prior probability set to 0 to ensure that prior is determined from training data and other parameters is various smoothing we use it with the default value of 1e-9 to avoid the numerical instability.

**Fig 13:**
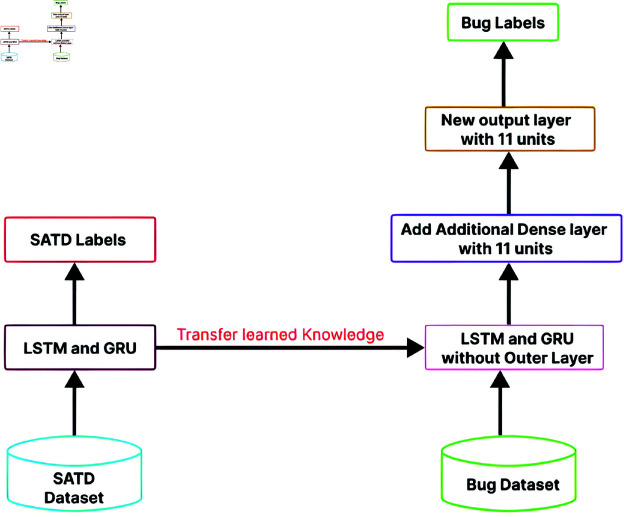
The image Illustrate Transfer learning process for deep learning models.

For the deep learning model, the process of transfer learning approach is shown in Fig 13.

LSTM and GRU were initially trained on the SATD dataset and learned patterns to identify and classify SATD effectively. These models captured complex patterns and relationships in data specific to SATD identification and classification. The knowledge (learned weights and patterns) from pre-trained LSTM and GRU models on the SATD dataset is transferred to the new task of Bug identification and classification. This step involved using the core architecture of the pre-trained LSTM and GRU models, but with some modifications to adapt them to the new task. Modification involves removing the outer layer, which was specific to the SATD labels; this layer is replaced to match the requirements of the new task, bug identification, and classification. An additional dance layer with 11 units is added; these layers are designed to capture task-specific patterns in the bug dataset. The parameters for these models include: The Adam optimizer is used to update the model weights, dropout layers with the rate of 0.5 were integrated to mitigate overfitting by randomly dropping some units during training, early stopping is also implemented during training when validation loss ceased improving, thus saving computational resource attention mechanism is also utilized. The batch size is the same as SATD, which is 128, and the number of epochs for both models is 8. A learning rate of 0.01 is added same as SATD.

**Fig 14:**
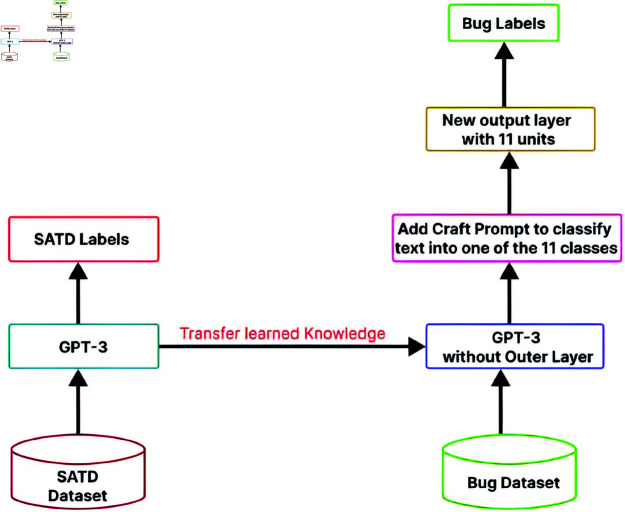
The image Illustrated Transfer learning process for Transformer Model.

The transfer learning approach for GPT-3 is shown in Fig 14.

GPT-3 model was initially trained on the SATD dataset; the model captured complex patterns and relationships in the data specific to SATD classification. The knowledge (learned weights and patterns) from the pre-trained GPT-3 models on the SATD dataset is transferred to the new task of Bug identification and classification. This process involves using the core architecture of GPT-3 but with some modifications to adapt GPT-3 to the new task. The outer layer of the GPT 3 model, which was specific to SATD labels, is removed; this layer is replaced to match the requirement of a new task, i.e., bug identification and classification. The core layers of the pre-trained model froze during the training on the new task. The weights of freezing layers are not updated during backpropagation. Another modification involves using Craft prompts that clearly instruct GPT-3 to generate or classify text into one of the 11 classes. We also set the temperature to lower 0.1 for better classification. The parameters setting of the model includes dropout layers with a rate of 0.1 were integrated to mitigate the overfitting problem by randomly dropping the units during the training, early stopping with the patience of 2 is also implemented to halt the training when the validation loss stops improvement it prevents overfitting and saving the computational resources, the batch size is same as SATD which is 32, number of epochs is 5 and the learning rate 1e - 5 is added same as SATD.

### 5.5 Model evaluation

These metrics—Accuracy, Precision, Recall, and F1 score—along with the classification report, confusion matrix, and trained models were used for evaluation:

**Precision:** The ratio of the number of positive observations that were actually observed to the total number of positive observations that were expected is called precision.

$$\text{Precision}=\frac{\text{True Positives}}{\text{True Positives}+\text{False Positives}}$$ (6)

Where:

The number of correctly detected positive observations is represented by True Positives. A false positive is the number of incorrectly predicted positive observations.

10. **Recall:** Recall is defined as the proportion of accurately predicted positive instances relative to the total number of instances in the actual class.

$$\text{Recall}=\frac{\text{True Positives}}{\text{True Positives}+\text{False Negatives}}$$ (7)

Where:

False negatives refer to the quantity of observations that are incorrectly predicted as negative. **Accuracy:** When compared to the total number of observations, accuracy is the percentage of predictions that were right.

$$\text{Accuracy}=\frac{\text{TP}+\text{TN}}{\text{TP}+\text{TN}+\text{FP}+\text{FN}}$$ (8)

Where:

TP means: (True Positives) denotes the count of accurately predicted positive instances. TN means: (True Negative) denotes the count of inaccurately predicted negative observations.

FP means: (False Positive) denotes the quantity of inaccurately predicted positive observations. FN means: (False Negative) denotes the count of inaccurately predicted negative observations.

11. **F1 Score:** F1-Score is the weighted average of precision and recall; it is considered both false positive and false negative.

$$\text{F1 Score}=2\times \frac{\text{Precision}\times \text{Recall}}{\text{Precision}+\text{Recall}}$$ (9)

## 6 Results

In this section, we first present our experimental results, and then present the comparative analysis for both SATD and Bugs identification and classification. After this, we show our key findings and lastly answer how we addressed our research questions based on our experimental results and key findings.

### 6.1 Self-admitted technical debt results

In this section, we present the results of our experiment on identifying and classifying the SATD using machine learning models as baseline models, Deep learning models, and transformer models. The models were trained and tested on the SATD dataset. We evaluate the performance of each model using accuracy, precision, recall, and F1-score.

#### 6.1.1 Baseline models.

The performance of baseline models, i.e., naive Bayes and AdaBoost classifiers, was evaluated using the above-mentioned performance metrics. The Results are shown in below Tables 5 and 6.

**Table 5:** Detail of precision, recall, accuracy, and F1 Score for Naïve Bayes on SATD.

Class	Precision	Recall	F1-Score	Support
0	0.83	0.81	0.82	3952
1	1.00	0.94	0.97	3952
2	0.94	0.92	0.93	3952
3	1.00	0.84	0.92	3952
4	0.87	0.97	0.91	3952
5	0.97	0.96	0.96	3952
6	0.60	0.73	0.66	3952
7	0.89	0.94	0.92	3952
8	0.99	0.87	0.93	3952
**Accuracy**				0.89
**Macro Avgerage**	0.90	0.89	0.89	35568
**Weighted Avgerage**	0.90	0.89	0.89	35568

**Table 6:** Detail of precision, recall, accuracy, and F1 Score for AdaBoost Classifier on SATD.

Class	Precision	Recall	F1-Score	Support
0	0.98	0.88	0.93	3952
1	1.00	1.00	1.00	3952
2	0.97	0.94	0.96	3952
3	1.00	0.89	0.94	3952
4	0.99	0.86	0.92	3952
5	1.00	0.84	0.91	3952
6	0.42	0.92	0.58	3952
7	1.00	0.65	0.78	3952
8	0.97	0.72	0.83	3952
**Accuracy**				0.85
**Macro Avg**	0.92	0.86	0.87	35568
**Weighted Avg**	0.93	0.85	0.87	35568

**Fig 15:**
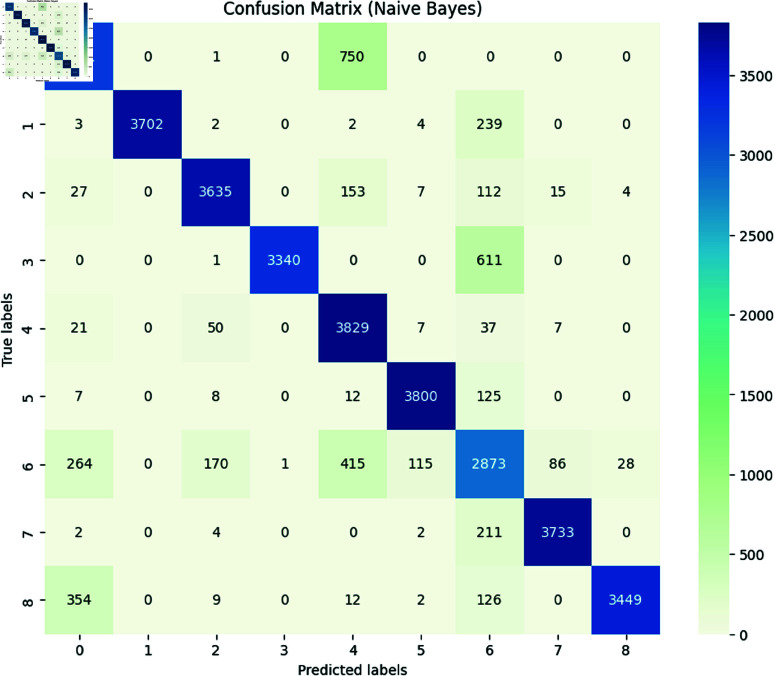
The image Illustrated the Confusion Matrix provides a detailed summary of the performance of a naïve Bayes model by presenting the counts of true positives (TP), true negatives (TN), false positives (FP), and false negatives (FN). The rows correspond to the actual classes, while the columns represent the predicted classes. Correct predictions are reflected along the diagonal elements, whereas the off-diagonal elements indicate the instances of misclassification.

**Fig 16:**
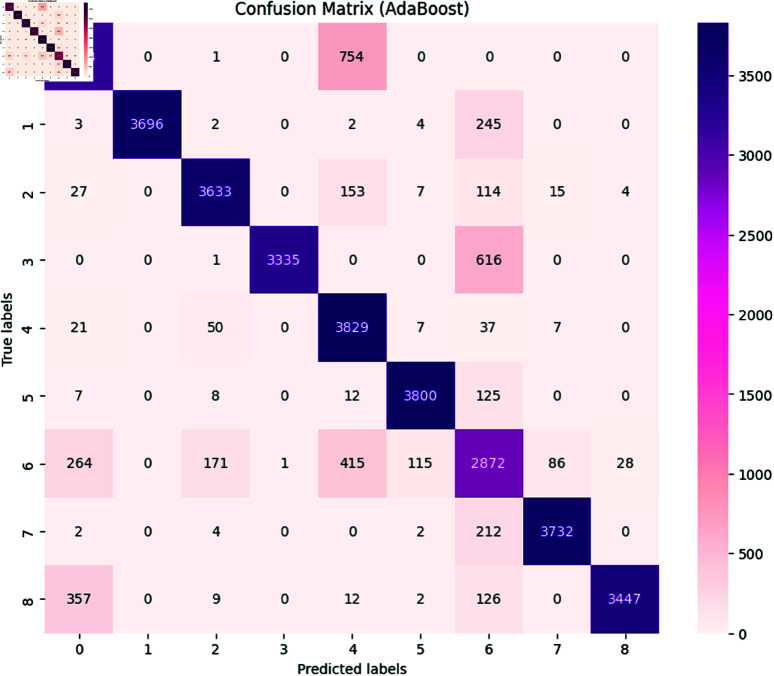
The image Illustrated the Confusion Matrix displays the quantities of true positives (TP), true negatives (TN), false positives (FP), and false negatives (FN) generated by the AdaBoost Classifier model. The rows denote the actual classes, whereas the columns indicate the predicted classes. The diagonal elements represent the count of accurate predictions, whereas the off-diagonal elements reflect instances of misclassification.

1. ***Naïve Bayes:*** The Naive Bayes achieved an accuracy of 0.89 with detailed performance metrics shown in Table 5 and a confusion matrix shown in Fig 15. The classification report illustrated the ability of models to identify and classify the several types of SATD in comments. As we mentioned in Sect 4, the classes represent the specific type of SATD: Non-debt, design debt, documentation debt, code debt, architecture debt, test debt, requirement debt, build debt, and defect debt. The classification report shows that the model achieved high precision, recall, and F1-score in class 0, which means that the model can effectively identify the comments with no SATD and classify them as non-debt. The model also achieves high precision, recall, and F1-score in all the classes except class 6, which represents requirements debt. In this class model has low precision, recall and F1 score, indicating that the model can identify SATD but has difficulty in correctly classifying requirements debt. The overall macro average for precision is 0.90, recall is 0.89, and the F1-score is 0.89, which indicates that the model performs well across most SATD categories, however, the classification of requirements debt is challenging, and models tend to confuse it with other SATD.
2. **Adaboost Classifier** The Adaboost Classifier achieved an accuracy of 0.85 with detailed performance metrics shown in table and confusion matrix shown in Fig 16. The classification report illustrated the ability of models to identify and classify the distinct types of SATD in comments. As we mentioned in Sect 4 the classes represent the specific type of SATD: Non-debt, design debt, documentation debt, code debt, architecture debt, test debt, requirement debt, build debt, and defect debt. The classification report indicates that the model achieved high precision, recall, and F1-score in class 0, which means that the model can effectively identify the comments that do not contain SATD and classify them as non-debt. The model also achieves high precision, recall, and F1-score in all the classes except class 6, which represents requirements debt, and class 7, which represents build debt. In class 6 model had low precision, indicating that the model identifies comments as requirement debt but is incorrect in its classification. In class 7 model has a lower recall, which indicates that the model accurately identifies built debt, but it misses many instances. The overall macro average for precision is 0.92, recall is 0.86 and the F1-score is 0.87. This indicates that the model performs well across most SATD categories, however, the classification of requirements debt and build debt is challenging.

#### 6.1.2 Deep learning models.

**Fig 17:**
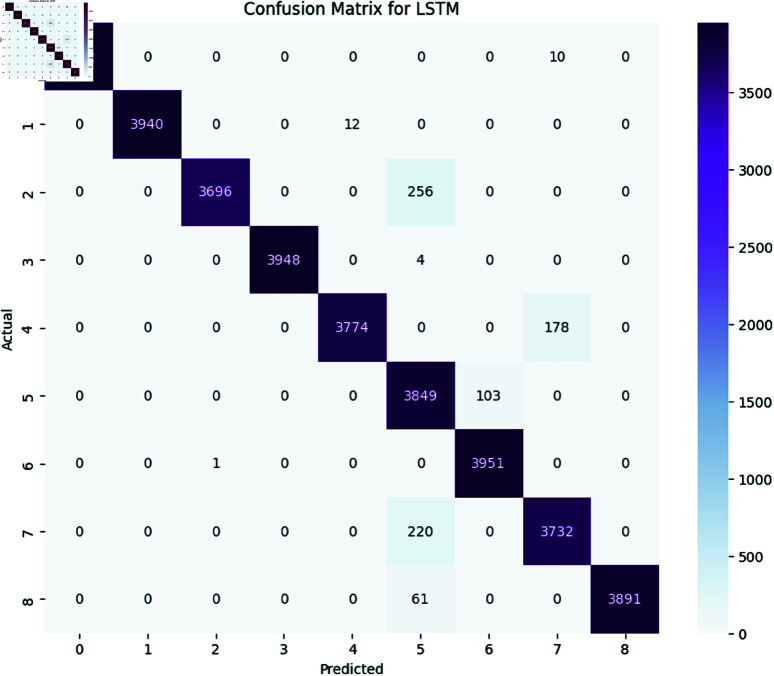
The image Illustrated the Confusion Matrix displays the quantities of true positives (TP), true negatives (TN), false positives (FP), and false negatives (FN) predicted by the LSTM. The rows denote the actual classes, whilst the columns signify the predicted classes. The diagonal elements represent the quantity of accurate predictions, and the off-diagonal elements denote misclassifications.

**Fig 18:**
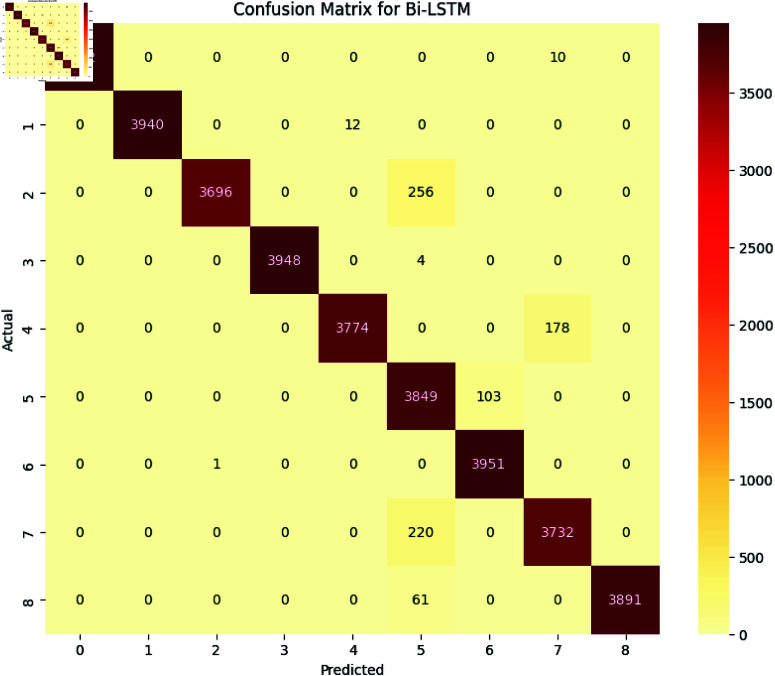
The image Illustrated the Confusion Matrix displays the quantities of true positives (TP), true negatives (TN), false positives (FP), and false negatives (FN) predicted by the LSTM. The rows denote the actual classes, whilst the columns signify the predicted classes. The diagonal elements represent the quantity of accurate predictions, and the off-diagonal elements denote misclassifications.

**Fig 19:**
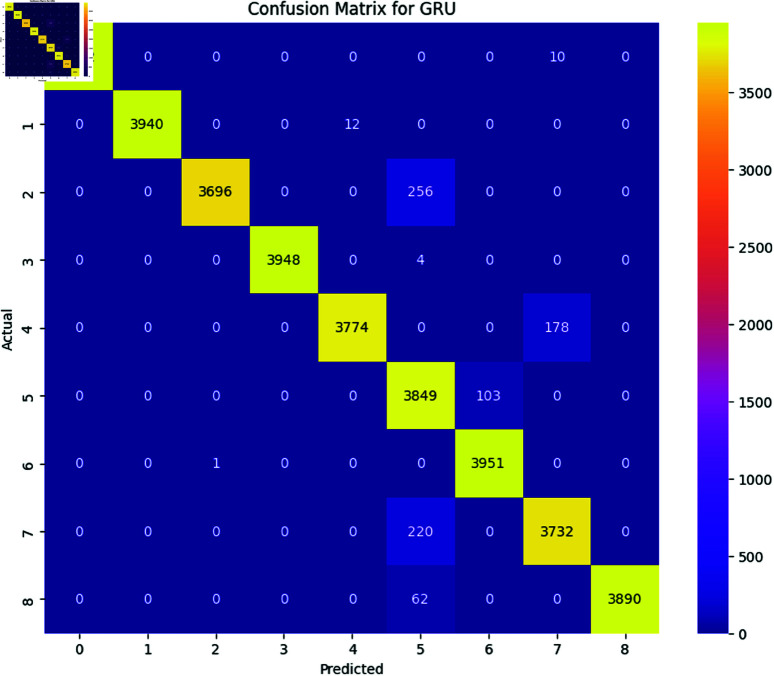
The image Illustrated the Confusion Matrix displays the quantities of true positive (TP), true negative (TN), false positive (FP), and false negative (FN) predictions generated by the GRU. The rows denote the actual classes, whereas the columns indicate the predicted classes. The diagonal elements represent the count of accurate predictions, whereas the off-diagonal elements illustrate instances of misclassification.

**Fig 20:**
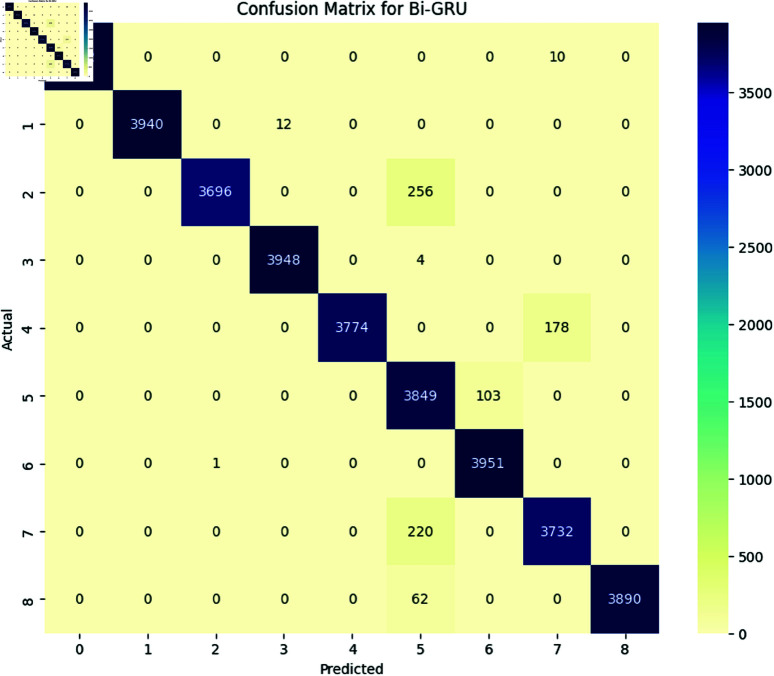
The image Illustrated the Confusion Matrix displays the quantities of true positives (TP), true negatives (TN), false positives (FP), and false negatives (FN) predicted by the BI-GRU. The rows denote the actual classes, whilst the columns signify the predicted classes. The diagonal elements represent the count of accurate predictions, and the off-diagonal elements denote misclassifications.

The performance of deep learning models, including LSTM, BiLSTM, GRU, and BiGRU, was assessed using several evaluation metrics, namely accuracy, precision, recall, and F1-Score. Additionally, the confusion matrix for each model is provided to offer a detailed view of their classification performance Figs 17, 18, 19, and 20.

All the deep learning models achieved an accuracy of 98 and the macro average for precision recall and F1-Score all are 0.98 which indicates that all deep learning models perform well across all the SATD categories. The High Performance across all categories except class 6 which represents requirements debt indicates that all deep learning models are highly effective in identifying and classifying SATD in code comments while class 6 has slightly low precision which indicates that the model accurately identifies all the requirements debt, but it occasionally misclassified some comments. Below Tables 7, 8, 9, and 10 represent detailed performance metrics of all deep learning models separately.

**Table 7:** Detail of precision, recall, accuracy, and F1 Score for LSTM on SATD.

Class	Precision	Recall	F1-Score	Support
0	1.00	1.00	1.00	3952
1	1.00	1.00	1.00	3952
2	1.00	0.94	0.97	3952
3	1.00	1.00	1.00	3952
4	1.00	0.96	0.98	3952
5	1.00	0.97	0.99	3952
6	0.82	1.00	0.90	3952
7	1.00	0.94	0.97	3952
8	1.00	0.98	0.99	3952
**Accuracy**				0.98
**Macro Avg**	0.98	0.98	0.98	35568
**Weighted Avg**	0.98	0.98	0.98	35568

**Table 8:** Detail of precision, recall, accuracy, and F1 Score for Bi-LSTM on SATD.

Class	Precision	Recall	F1-Score	Support
0	1.00	1.00	1.00	3952
1	1.00	1.00	1.00	3952
2	1.00	0.94	0.97	3952
3	1.00	1.00	1.00	3952
4	1.00	0.96	0.98	3952
5	1.00	0.97	0.99	3952
6	0.82	1.00	0.90	3952
7	1.00	0.94	0.97	3952
8	1.00	0.98	0.99	3952
**Accuracy**				0.98
**Macro Avg**	0.98	0.98	0.98	35568
**Weighted Avg**	0.98	0.98	0.98	35568

**Table 9:** Detail of precision, recall, accuracy, and F1 Score for GRU on SATD.

Class	Precision	Recall	F1-Score	Support
0	1.00	1.00	1.00	3952
1	1.00	1.00	1.00	3952
2	1.00	0.94	0.97	3952
3	1.00	1.00	1.00	3952
4	1.00	0.96	0.98	3952
5	1.00	0.97	0.99	3952
6	0.82	1.00	0.90	3952
7	1.00	0.94	0.97	3952
8	1.00	0.98	0.99	3952
**Accuracy**				0.98
**Macro Avg**	0.98	0.98	0.98	35568
**Weighted Avg**	0.98	0.98	0.98	35568

**Table 10:** Detail of precision, recall, accuracy, and F1 Score for Bi-GRU on SATD.

Class	Precision	Recall	F1-Score	Support
0	1.00	1.00	1.00	3952
1	1.00	1.00	1.00	3952
2	1.00	0.94	0.97	3952
3	1.00	1.00	1.00	3952
4	1.00	0.96	0.98	3952
5	1.00	0.97	0.99	3952
6	0.82	1.00	0.90	3952
7	1.00	0.94	0.97	3952
8	1.00	0.98	0.99	3952
**Accuracy**				0.98
**Macro Avg**	0.98	0.98	0.98	35568
**Weighted Avg**	0.98	0.98	0.98	35568

#### 6.1.3 Transformer models.

The performance of transformers models i.e., BERT and GPT-3 was evaluated using performance metrics accuracy, precision, recall and F1-Score. The results with detailed performance metrics are shown below in Tables 11 and 12.

**Table 11:** Detail of precision, recall, accuracy, and F1 Score for BERT on SATD.

Class	Precision	Recall	F1-Score	Support
0	1.00	1.00	1.00	3952
1	1.00	1.00	1.00	3952
2	1.00	0.94	0.97	3952
3	1.00	1.00	1.00	3952
4	1.00	0.96	0.98	3952
5	1.00	0.97	0.99	3952
6	0.84	1.00	0.90	3952
7	1.00	0.95	0.97	3952
8	1.00	0.98	0.99	3952
**Accuracy**				0.983
**Macro Avg**	0.981	0.982	0.981	35568
**Weighted Avg**	0.98	0.982	0.981	35568

**Table 12:** Detail of precision, recall, accuracy and F1 Score for GPT-3 on SATD.

Class	Precision	Recall	F1-Score	Support
0	1.00	1.00	1.00	3952
1	1.00	1.00	1.00	3952
2	1.00	0.94	0.97	3952
3	1.00	1.00	1.00	3952
4	1.00	0.96	0.98	3952
5	1.00	0.97	0.99	3952
6	0.84	1.00	0.90	3952
7	1.00	0.95	0.97	3952
8	1.00	0.98	0.99	3952
**Accuracy**				0.984
**Macro Avg**	0.981	0.983	0.982	35568
**Weighted Avg**	0.981	0.983	0.982	35568

**Fig 21:**
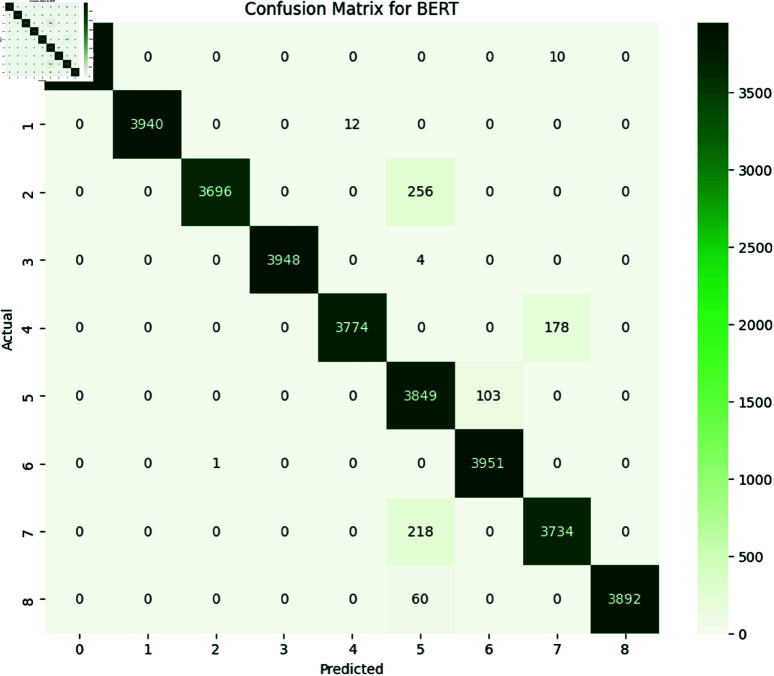
The image Illustrated the Confusion Matrix displays the counts of true positives (TP), true negatives (TN), false positives (FP), and false negatives (FN) predicted by BERT. The rows denote the actual classes, whilst the columns signify the predicted classes. The diagonal elements represent the count of accurate predictions, and the off-diagonal elements denote misclassifications.

**Fig 22:**
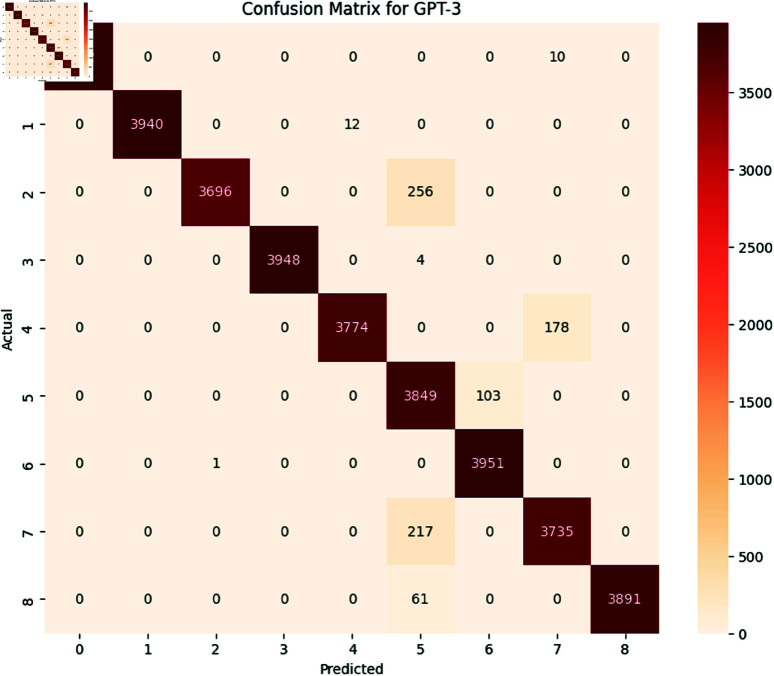
The image Illustrated the Confusion Matrix displays the quantities of true positives (TP), true negatives (TN), false positives (FP), and false negatives (FN) predicted by GPT-3. The rows denote the actual classes, whilst the columns indicate the anticipated classes. The diagonal elements represent the count of accurate predictions, and the off-diagonal elements denote misclassifications.

1. ***BERT (Bidirectional Encoder Representations from Transformers):***
  The BERT model achieved an overall accuracy of 98.3 The detailed performance metrics shown in Table 11 provide insight into the model’s capability of identifying and classifying SATD in code comments. The confusion matrix is shown below in Fig 21. As we already know, the classes represent the specific types of SATD. As we see in the classification report the model achieved high precision, recall, and F1-Score in all classes except class 6 which represents requirements debt this class model has slight precision which means the model identified requirements debt but misclassified some comments. Overall performance of BERT is high as the macro average for precision is 0.981, recall is 0.982 and F1-Score is 0.981 suggesting that the model is highly effective in both identifying and classifying SATD. The high levels of performance highlight the potential of BERT for SATD identification and classification tasks making it a valuable tool for software maintenance and quality assurance.
2. ***Generative Pre-trained Transformer (GPT-3)***
  GPT-3 model achieved an accuracy of 98.4. The detailed performance metrics shown in Table 12 provide insight into the model’s capability of identifying and classifying SATD in code comments. The confusion matrix is shown below in Fig 22. As we already know the classes represent the specific types of SATD. As we see in the classification report the model achieved high precision, recall, and F1-Score in all classes except class 6 which represents requirements debt this class model has slight precision which means the model identified requirements debt but misclassified some comments. Overall performance of GPT-3 is high as the macro average for precision is 0.981, recall is 0.983 and F1-Score is 0.982 suggesting that the model is highly effective in both identifying and classifying SATD. The high level of performance highlights the potential of BERT for SATD identification and classification tasks making it a valuable tool for software maintenance and quality assurance.

#### 6.1.4 Comparative analysis.

**Fig 23:**
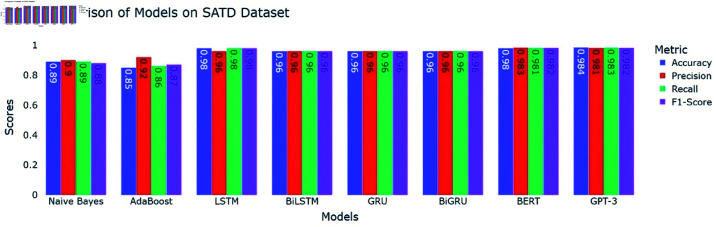
The image Illustrated Comparison of different models trained and evaluated on SATD.

Table 13 and Fig 23 reveal a significant performance difference between baseline models, deep learning models, and transformer models. The baseline model naive Bayes achieved accuracy and F1-Score 0.89 and the AdaBoost classifier achieved accuracy of 0.85 and F1-Score 0.87 these models provide a foundational benchmark demonstrating a reliable but limited performance on SATD identification and classification. In contrast, all the deep learning models achieved an accuracy of 0.98 and F1-Score of 0.98. This informality across deep learning models highlights their robustness and effectiveness in handling the complexity of SATD identification and classification. Transformer models BERT and GPT-3 further push the boundaries. The BERT achieved an average precision of 0.981, recall of 0.982, F1-Score 0.981, and overall accuracy is 0.983 and. GPT-3 achieved an average precision of 0.981, recall of 0.983, F1-Score 0.982, and overall accuracy is 0.984. The superior performance of deep learning models and transformers models makes them well suited for SATD identification and classification tasks.

**Table 13:** Comparative analysis of different models used for self-admitted technical identification and classification.

Model	Avg Precision	Avg Recall	Avg F1-Score	Accuracy
**Naïve Bayes**	0.90	0.89	0.89	0.89
**AdaBoost Classifier**	0.92	0.86	0.87	0.85
**LSTM**	0.98	0.98	0.98	0.98
**BiLSTM**	0.98	0.98	0.98	0.98
**GRU**	0.98	0.98	0.98	0.98
**BiGRU**	0.98	0.98	0.98	0.98
**BERT**	0.981	0.982	0.981	0.983
**GPT-3**	0.981	0.983	0.982	0.984

### 6.2 Bug dataset results

This section presents the results of our experiments on the identification and classification of bugs utilizing traditional machine learning, deep learning approaches, and transformer models. Naive Bayes was chosen as the representative machine learning model, while LSTM and GRU were selected as the deep learning models, and GPT-3 was designated as the Transformer model. The evaluation of these models was conducted using metrics such as accuracy, precision, recall, and F1-score. The classes exhibit the subsequent sequences: cdt-parser, deprecated7, cdt-build, cdt-core, cdt-debug, cdt-doc, cdt-indexer, cdt-debug-dsf, cdt-debug-dsf-gdb, cdt-build-managed, cdt-editor.

#### 6.2.1 Performance of pre-trained Naive Bayes.

**Fig 24:**
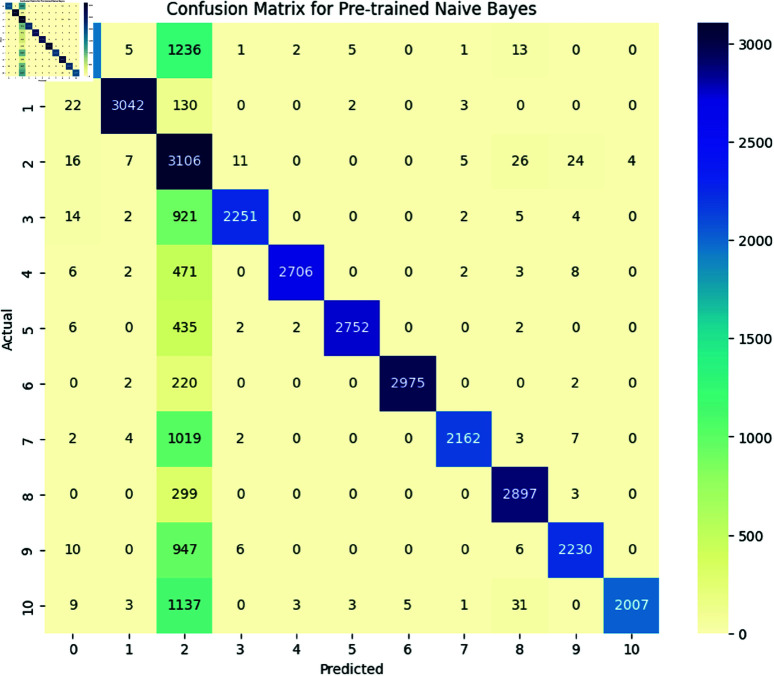
The image Illustrated the Confusion Matrix displays the quantities of true positives (TP), true negatives (TN), false positives (FP), and false negatives (FN) predicted by the pre-trained Naive Bayes model. The rows denote the actual classes, whilst the columns signify the predicted classes. The diagonal elements represent the count of accurate predictions, and the off-diagonal elements denote misclassifications.

The pre-trained Naïve Bayes model was fine-tuned and trained on the bug dataset; its performance metrics are summarized in Table 14 and confusion matrix shown in Fig 24. The overall macro average for precision is 0.85, recall is 0.82 and F1-Score is 0.83 respectively indicating that the model performs well across the entire component. The high performance across various categories suggests that the model is effective in both identifying and classifying bugs in different components with some room for improvement in categories like cdt- build (class 2). The pre-trained Naive Bayes model demonstrates reasonable performance with an overall accuracy of 82 the model is effective in identifying and classification the classes (e.g., deprecated7, cdt-indexer, and cdt-debug-dsf-gdb) but struggles with others (e.g., cdt-build, cdt-editor) the model result underscore the potential of using the transfer learning for bug identification and classification based on SATD.

**Table 14:** Detail of precision, recall, accuracy and F1 Score for pre-trained Naïve Bayes on Bug Dataset.

Class	Precision	Recall	F1-Score	Support
0	0.78	0.69	0.73	3199
1	0.98	0.96	0.97	3199
2	0.34	0.65	0.45	3199
3	0.89	0.78	0.83	3199
4	0.90	0.87	0.89	3199
5	0.94	0.86	0.89	3199
6	0.98	0.93	0.95	3199
7	0.95	0.88	0.91	3199
8	0.97	0.91	0.94	3199
8	0.97	0.91	0.94	3199
9	0.82	0.77	0.80	3199
10	0.84	0.71	0.77	3199
**Accuracy**				0.82
**Macro Avg**	0.85	0.82	0.83	35189
**Weighted Avg**	0.86	0.82	0.83	35189

### 6.3 Pre-trained LSTM performance

**Fig 25:**
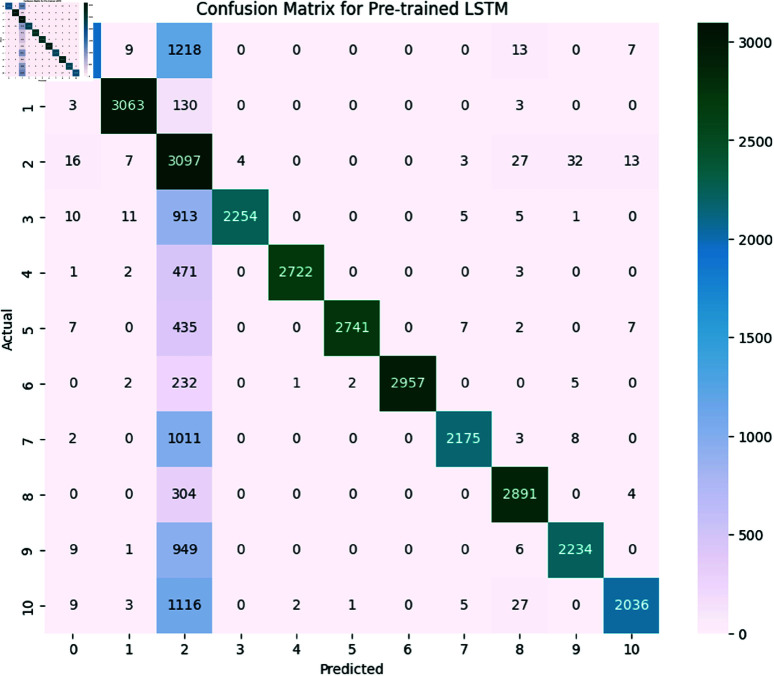
The image Illustrated the Confusion Matrix displays the quantities of true positives (TP), true negatives (TN), false positives (FP), and false negatives (FN) predicted by the Pre-trained LSTM. The rows denote the actual classes, whilst the columns signify the predicted classes. The diagonal elements represent the count of accurate predictions, and the off-diagonal elements denote misclassifications.

The performance metrics of pre-trained LSTM are Summarized in Table 15, and the confusion matrix is shown in Fig 25. The model achieved an overall accuracy of 80. As we see in the classification report the macro average for precision is 0.93, recall is 0.80 and F1-Score is 0.83. this indicates that the pre-trained LSTM model performs well across all the bug components the high performance across various categories suggests that the model is effective in both identifying and classifying the bug in different components, but some categories need improvement like class 2 (cdt- build) and class 10 (cdt-editor). The pre-trained LSTM model demonstrates reasonable performance, and the detailed metric highlights the model’s strengths and weaknesses guiding further improvement and fine-tuning, but this result highlights the potential of transfer learning for bug identification and classification based on SATD.

**Table 15:** Detail of precision, recall, accuracy and F1 Score for pre-trained LSTM on Bug Dataset.

Class	Precision	Recall	F1-Score	Support
0	0.97	0.61	0.75	3199
1	0.99	0.96	0.97	3199
2	0.32	0.97	0.48	3199
3	1.00	0.70	0.83	3199
4	1.00	0.85	0.92	3199
5	1.00	0.86	0.92	3199
6	1.00	0.92	0.96	3199
7	0.99	0.68	0.81	3199
8	0.97	0.90	0.94	3199
9	0.98	0.70	0.82	3199
10	0.98	0.64	0.77	3199
**Accuracy**				0.80
**Macro Avg**	0.93	0.80	0.83	35189
**Weighted Avg**	0.93	0.80	0.83	35189

#### 6.3.1 Performance of pre-trained GRU.

**Fig 26:**
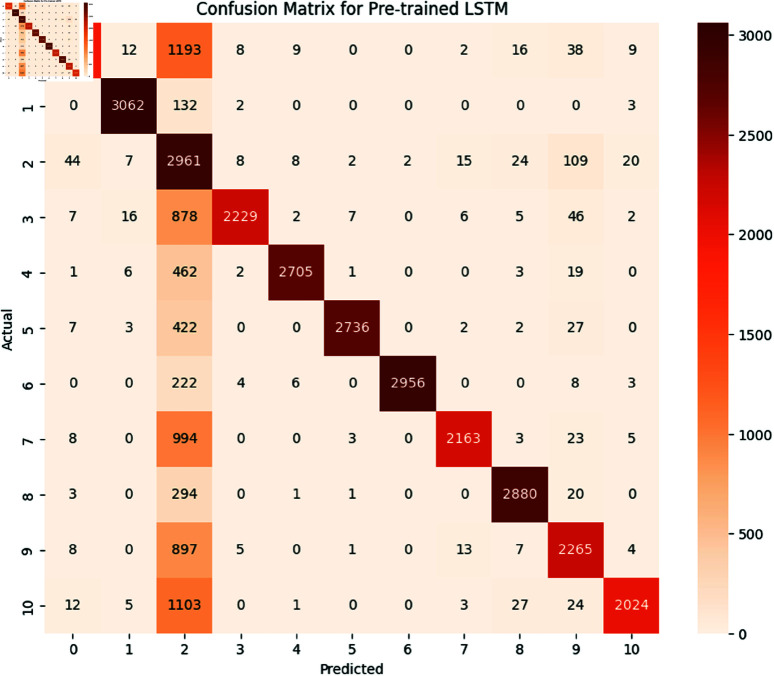
The image Illustrated the Confusion Matrix displays the quantities of true positives (TP), true negatives (TN), false positives (FP), and false negatives (FN) predicted by the Pre-trained GRU. The rows denote the actual classes, whilst the columns signify the predicted classes. The diagonal elements represent the count of accurate predictions, and the off-diagonal elements denote misclassifications.

The pre-trained GRU fine-tuned and trained on the bug dataset and achieved an overall accuracy of 0.81. The detailed performance metrics are summarized in Table 16 and the confusion matrix is shown in Fig 26. The overall macro average for precision is 0.91, recall is 0.80 and F1-Score is 82 which models effectiveness in identifying and classification the bugs based on SATD. The high performance across various categories (e.g., deprecated7, cdt-indexer, cdt-debug-dsf-gdb) but struggles with others (e.g., cdt-build, cdt-parser) suggests that the model is effective in identifying and classifying bugs into different components and model is not perform well in some categories like cdt-build and cdt-parser (class 0 and class 2). The results underscore the potential of using transfer learning for bug identification and classification based on SATD, but some could improve for better accuracy and reliability.

**Table 16:** Detail of precision, recall, accuracy and F1 Score for pre-trained GRU on Bug Dataset.

Class	Precision	Recall	F1-Score	Support
0	0.95	0.60	0.73	3199
1	0.98	0.96	0.97	3199
2	0.35	0.93	0.47	3199
3	0.99	0.72	0.82	3199
4	0.99	0.85	0.91	3199
5	0.99	0.86	0.92	3199
6	1.00	0.92	0.96	3199
7	0.98	0.68	0.80	3199
8	0.97	0.90	0.93	3199
9	0.88	0.71	0.79	3199
10	0.98	0.63	0.77	3199
**Accuracy**				0.81
**Macro Avg**	0.91	0.80	0.82	35189
**Weighted Avg**	0.91	0.80	0.82	35189

#### 6.3.2 Performance of pre-trained GPT-3.

**Fig 27:**
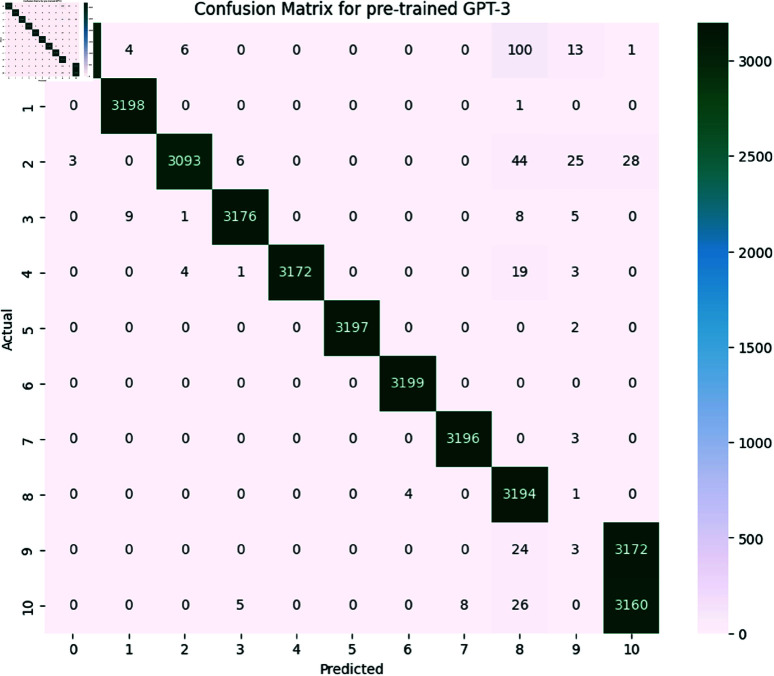
The image Illustrated the Confusion Matrix displays the quantities of true positives (TP), true negatives (TN), false positives (FP), and false negatives (FN) predicted by the Pre-trained GPT-3. The rows denote the actual classes, whilst the columns signify the predicted classes. The diagonal elements represent the count of accurate predictions, and the off-diagonal elements denote misclassifications.

The pre-trained GPT-3 fine-tuned on the bug dataset, and the model achieved an overall accuracy of 0.96. The performance metrics are summarized in Table 17, and the confusion matrix is shown in Fig 27. The detailed performance metrics from the classification report offer insights into model effectiveness in identifying and classifying several components of bugs based on SATD. The overall macro average for precision is 0.96, recall is 0.96, and F1-Score is 0.96, indicating that GPT-3 performs well across all the bug components. The high performance across various categories suggests that the model is effective in both identifying and classifying the bugs into different components, with some room for improvement in categories like cdt-build (class2). The model achieved an overall accuracy of 0.92. This means that models are effective in identifying and classifying most of the classes, but struggle with some classes like cdt-build. The result underscores the potential of using transfer learning in transformer models for bug identification and classification.

**Table 17:** Detail of precision, recall, accuracy and F1 Score for pre-trained GPT-3 on Bug Dataset.

Class	Precision	Recall	F1-Score	Support
0	0.96	0.92	0.94	3199
1	1.00	0.99	0.99	3199
2	0.83	0.92	0.87	3199
3	0.98	0.96	0.97	3199
4	0.99	0.97	0.98	3199
5	0.99	0.98	0.99	3199
6	1.00	1.00	1.00	3199
7	0.93	0.97	0.95	3199
8	0.97	0.98	0.98	3199
9	0.97	0.94	0.96	3199
10	0.98	0.94	0.96	3199
Accuracy				0.96
Macro Avg	0.96	0.96	0.96	35189
Weighted Avg	0.96	0.96	0.96	35189

#### 6.3.3 Comparative analysis.

**Fig 28:**
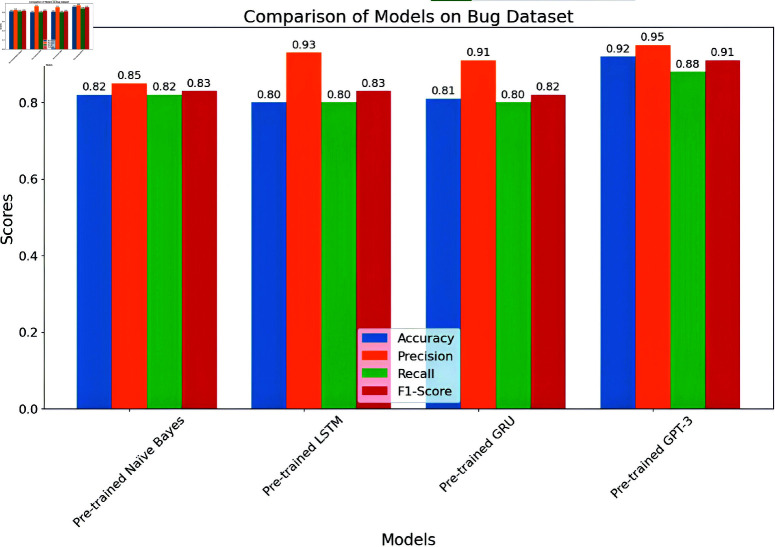
The image Illustrated Comparison of different models trained and evaluated on Bug Dataset.

Table 18 and Fig 28 reveal that the pre-trained GPT-3 model outperformed the others and achieved an average precision of 0.96, average recall of 0.96, average F1 score of 0.96 and accuracy of 0.96. This indicates that GPT-3 has superior capability in identifying and classifying bugs. The pre-trained Naive Bayes models serving as baseline models show a respectable performance with an average precision of 0.85, recall of 0.82, F1 score of 0.83, and accuracy of 0.82. The pre-trained LSTM and GRU both deep learning approaches showed comparable results, but LSTM slightly outperformed the GRU in terms of precision, but both showed an identical recall of 0.80. This analysis highlights the reasonable efficacy of the GPT 3 model exhibited the best overall performance for bug identification and classification using the transfer learning approach.

**Table 18:** Comparative analysis of different models used for bug identification and classification.

Model	Avg Precision	Avg Recall	Avg F1-Score	Accuracy
**Pre-trained Naïve Bayes**	0.85	0.82	0.83	0.82
**Pre-trained LSTM**	0.93	0.80	0.83	0.80
**Pre-trained GRU**	0.91	0.80	0.82	0.81
**Pre-trained GPT-3**	0.96	0.96	0.96	0.96

#### 6.3.4 Comparative analysis with existing approaches.

To validate the significance of our proposed approach, we compared our system’s performance with existing SATD and bug detection models, including SATDID [18], DeepCom [21], and BUGLAB [20]. Our unified deep learning model demonstrated higher accuracy, F1 score, and precision, particularly in handling imbalanced classes such as requirement debt and build debt. While existing methods like DeepCom are limited to comment generation and SATDID focuses solely on SATD without integrating bug analysis, our approach bridges this gap by effectively leveraging transformer models to address both issues. The comparative results highlight that our methodology significantly outperforms traditional machine learning baselines and recent SATD-specific systems, proving its robustness and applicability in real-world scenarios.

## 7 Discussion

### 7.1 Research contribution of proposed work

As we investigate our research questions in Sect 5.1, we now present how we can address these questions based on experimental results and key findings.

12. RQ1 is designed to explore the feasibility and method of creating a unified approach to simultaneously identify SATD and bugs by analyzing the comments associated with code and classifying them. We addressed this question by leveraging transfer learning and developed an integrated approach where models are trained on the SATD dataset and fine-tuned for bug detection and classification. This approach allows for the simultaneous analysis of comments related to code, utilizing models like GPT-3, which has been shown to effectively identify and classify bugs with high accuracy. This approach helps the developers to maintain code quality and effectively manage SATD.

13. RQ2 is designed to investigate the performance of deep learning for identifying SATD and detecting Bugs and classifying them as compared to baseline models, i.e., traditional machine learning models. These questions are addressed based on experimental results. As we show in our experimental result, the deep learning models demonstrate high effectiveness with an accuracy and F1 score of 0.98 on the SATD dataset. On the bug dataset, the pre-trained LSTM and GRU also show reasonable performance with light variance, confirming that these advanced neural network architectures learn complex patterns and contextual information.

14. RQ3 is designed to explore the role of NLP techniques in analyzing and processing the comments associated with code. This question is also addressed based on experimental results, The NLP techniques (removal of stop words, Stemming, and lemmatization) implemented in transformer models like BERT and GPT-3 were effectively applied to analyze the comments associated with code. These models capture the semantics and syntactic nuances of comments leading to enhanced performance in both SATD and bug identification and classification, as evidenced by their superior metrics compared to baseline models and deep learning models. But in SATD the NLP techniques applied on deep learning models can also help to analyze the comments associated with code. By enhancing the SATD identification and Detection through improved comment analysis, this research led to more accurate tools for software maintenance.

15. RQ4 is designed to focus on comprehensive testing and proposed integrated approach and assess the performance and effectiveness of the approach across diverse datasets. As we have shown extensive experimentation reveals that transformer models particularly GPT-3 achieve the highest performance metrics across both datasets the result underscores the visibility and effectiveness of the purpose-integrated approach of transfer learning this approach significantly improves the accuracy and effectiveness of identifying and classifying SATD and bugs providing a reliable tool for software maintenance and quality insurance across a divorce software project and datasets.

### 7.2 Research impact

This research presents a pioneering approach to identifying software defects and self-admitted technical debt (SATD) by applying deep learning techniques, specifically GRU and BiGRU architectures. The study holds substantial significance for both academic research and practical software engineering applications. Firstly, it addresses a persistent challenge in the software development lifecycle: the early and accurate identification of technical debt and defect-prone code regions. SATD, often introduced through developer comments as shortcuts or temporary fixes, can severely degrade maintainability and increase long-term costs if not detected and managed promptly. By leveraging deep learning, this study automates the classification of SATD and defects from source code comments, thereby reducing the manual effort and potential biases involved in traditional detection approaches. The integration of GRU and BiGRU models introduces a novel angle, as these architectures are well-suited to capturing the sequential dependencies and contextual nuances within source code comments. The bidirectional nature of BiGRU, in particular, allows for a comprehensive understanding of both preceding and succeeding textual context, which significantly enhances classification accuracy compared to traditional machine learning models and unidirectional LSTMs. Experimentally, the study is validated on real-world, open-source datasets, demonstrating its robustness and generalizability. The deep learning models consistently outperform existing baseline techniques in detecting SATD and defects, showing higher precision, recall, and F1 scores. This evidences the potential for integrating such models into developer workflows or automated code review systems. Beyond the technical advancements, this research contributes to the broader goal of building more sustainable and maintainable software systems. By automating SATD and defect detection, software teams can more proactively manage code quality, mitigate risks earlier in the development cycle, and ultimately improve long-term software health.

In summary, the study not only enriches the academic discourse around AI-driven software analysis but also lays the groundwork for practical tools that can be adopted in industry. It encourages further exploration into deep learning applications in software engineering, particularly in areas such as refactoring recommendation, intelligent code review, and maintainability prediction.

### 7.3 Key findings

1. Deep learning models (LSTM, BI-LSTM, GRU, and BI-GRU) and transformer models (BERT and GPT-3 significantly outperformed the baseline models in identifying and classifying SATD. All deep learning models achieved an accuracy and F1 score of 0.98, while BERT and GPT-3 had slightly higher metrics, with GPT-3 reaching an overall accuracy of 0.984. This demonstrates the robustness and effectiveness of advanced neural network architecture in handling the complexity of SATD identification and classification.
2. The transfer learning approach on the bug dataset shows that the pre-trained GPT-3 model outperformed the other in bug identification and classification. The GPT-3 model achieved an average precision of 0.95, recall of 0.88, F1 score of 0.91, and accuracy of 0.92. This indicates the strong capability of the GPT-3 model in transfer learning, highlighting its adaptability and superior performance compared to other models.
3. The baseline models, Naïve Bayes and AdaBoost classifier, provide a foundational Benchmark for SATD, and pre-trained Naïve Bayes provides the benchmark for the bug dataset. Although they showed respectable performance with accuracy and F1-score range from 0.82 to 0.89. This indicates that while baseline models can provide a starting point, more advanced models are necessary for high accuracy and effective identification and classification tasks.

### 7.4 Significance of the study

The proposed framework represents a substantial advancement in the fields of software engineering and automated maintenance. By concurrently identifying SATD and software bugs using advanced deep learning and NLP techniques, our system enhances the software development life cycle in the following ways:

- Early and accurate detection of technical debt and bugs contributes to cleaner, more maintainable code.
- Automating these tasks reduces the time and resources developers spend on manual analysis.
- The system can be integrated into modern CI/CD pipelines for real-time analysis, providing practical benefits to software teams.
- Our integration of transfer learning for SATD-bug tasks opens avenues for further exploration in cross-domain defect analysis.

## 8 Threats to validity and limitations

We acknowledge the following limitations and potential threats to validity in our study:

1. The quality of our results depends on the labeled datasets used. Some manually annotated SATD or bug instances may be prone to bias or inconsistencies, affecting training accuracy.
2. Our approach is trained on open-source projects (e.g., Eclipse, Mozilla, Apache). While diverse, these datasets may not fully represent proprietary software systems or other languages beyond English.
3. Transformer models such as GPT-3 require substantial computational resources and may not be feasible for small teams or real-time deployment without optimization.
4. Despite applying different techniques to balance the datasets, class imbalance may still influence the generalization of some underrepresented categories (e.g., requirement or build debt).

Acknowledging these threats helps contextualize the findings and guides future enhancements of the proposed approach.

## 9 Conclusion and future work

In this study, we aimed to develop an integrated approach that simultaneously identifies and classifies self-admitted technical debt and identifies and classifies bugs based on SATD by using advanced neural network architecture and transformer models and shows that these advanced neural network architectures outperform the traditional machine learning models serving as our baseline models in handling the complexity of these tasks. Our key finding supports this as we successfully develop an integrated approach using transfer learning that identifies and classifies the SATD and bugs simultaneously and we also see that deep learning models (LSTM, BI LSTM, GRU, and BI GRU) and transformers models (BERT and GPT-3) outperformed the baseline models (Naive Bayes and Ad boost classifier) in identifying and classifying SATD. All deep learning models achieved an accuracy and precision of 0.98, and transformer models achieved slightly higher metrics. The GPT-3 achieved an overall accuracy of 0.984. We see that using the transfer learning approach the transformer model (GPT-3) outperformed the other as it achieved an overall accuracy of 0.96 and F1-Score of 0.96, precision of 0.96, and recall of 0.96, and deep learning models (LSTM, GRU) also give significant performance, but their accuracy is slightly lower than baseline model (Naive Bayes). The significance and implications of these findings are profound, they demonstrate the robustness and effectiveness of advanced neural network architecture in managing complex textual data in software engineering tasks. Our results underscore the potential of deep learning models and transformer models to significantly enhance the identification and classification of SATD and bugs, which are critical for maintaining software quality and reducing technical debt. Although we effectively created a cohesive strategy for both challenges and attained notable performance with sophisticated neural network architecture, there remain several opportunities for future exploration. In the future, we will augment the dataset to encompass a broader array of applications and a more diversified range of SATD and faults. Furthermore, we enhanced data quality for bug discovery and classification, hence augmenting the performance of deep learning and transformer models. We will devise a methodology to augment the explainability and interpretability of models, facilitating a deeper comprehension of the decision-making process and aiding in the analysis of underlying data patterns. We also execute and evaluate the models in a real-time environment, which can yield useful insights and assist in analysing the models’ practicality and efficacy in real-world contexts.
